# Multiparametric Analyses Reveal the pH-Dependence of Silicon Biomineralization in Diatoms

**DOI:** 10.1371/journal.pone.0046722

**Published:** 2012-10-29

**Authors:** Vincent Hervé, Julien Derr, Stéphane Douady, Michelle Quinet, Lionel Moisan, Pascal Jean Lopez

**Affiliations:** 1 Biomineralisation et Morphogenèse, CNRS UMR-8189, Ecole Normale Supérieure, Paris, France; 2 Laboratoire Matière et Systèmes Complexes, CNRS UMR 7057, Université Paris Diderot, Paris, France; 3 Laboratoire Mathématiques Appliquées à Paris 5, CNRS UMR 8145, Université Paris Descartes, Paris, France; 4 Laboratoire d'Excellence “CORAIL: Les récifs coralliens face au changement global,” Evolution des Biomineralisations, UMR CNRS 7208-MNHN-UPMC-IRD 207, Paris, France; Institute of Marine Research, Norway

## Abstract

Diatoms, the major contributors of the global biogenic silica cycle in modern oceans, account for about 40% of global marine primary productivity. They are an important component of the biological pump in the ocean, and their assemblage can be used as useful climate proxies; it is therefore critical to better understand the changes induced by environmental pH on their physiology, silicification capability and morphology. Here, we show that external pH influences cell growth of the ubiquitous diatom *Thalassiosira weissflogii*, and modifies intracellular silicic acid and biogenic silica contents per cell. Measurements at the single-cell level reveal that extracellular pH modifications lead to intracellular acidosis. To further understand how variations of the acid-base balance affect silicon metabolism and theca formation, we developed novel imaging techniques to measure the dynamics of valve formation. We demonstrate that the kinetics of valve morphogenesis, at least in the early stages, depends on pH. Analytical modeling results suggest that acidic conditions alter the dynamics of the expansion of the vesicles within which silica polymerization occurs, and probably its internal pH. Morphological analysis of valve patterns reveals that acidification also reduces the dimension of the nanometric pores present on the valves, and concurrently overall valve porosity. Variations in the valve silica network seem to be more correlated to the dynamics and the regulation of the morphogenesis process than the silicon incorporation rate. These multiparametric analyses from single-cell to cell-population levels demonstrate that several higher-level processes are sensitive to the acid-base balance in diatoms, and its regulation is a key factor for the control of pattern formation and silicon metabolism.

## Introduction

Diatoms are a class of unicellular photosynthetic eukaryotes that can dominate marine and freshwater microalgal communities [1], [2]. They assimilate silicic acid, the soluble form of silicon, and use it to form their skeletons, named frustules. Frustules have been proposed to play an important role in diatoms. They can serve as a structural element for defense against predators [3], or be used to counter-balance turgor pressure or regulate exchanges with the environment [4]. They also act as an effective pH buffer [5], and protect cells against infection by parasites [6]. Therefore, disturbances in diatoms' ability to build up their siliceous skeleton is expected to have a large impact on cell growth, which can in turn impact the silicon cycle [7], [8]. Moreover, as diatoms can contribute to the formation of particle aggregates and frustules can function as ballast, disturbances of the frustule formation process can also modify the carbon export mechanism [9], [10], [11]. Alternatively, disturbances of diatom growth can also favor the growth of non-siliceous species of phytoplankton and therefore alter global primary productivity.

Recently, a few studies more specifically addressed the consequences of an increase in *p*CO_2_/decrease in pH on diatom physiology. Laboratory experiments showed that, as predicted by changes in iron chemistry, acidification from around 8.6 to 7.7, decreased iron uptake rates in two centric diatoms from the *Thalassiosira* genus and in one pennate species (*Phaeodactylum tricornutum*) [12]. Lowering the pH also decreased the proportion of total intracellular carbohydrate storage in *Chaetoceros muelleri*, and increased the amount of dissolved carbohydrate exudates [13]. Likewise, high CO_2_ combined with silicate or nitrate limitation increased production of the toxine domoic acid by *Pseudo-nitzschia* [14], [15]. Long-term adapted cultures (*ca.* 100 generations) of *Thalassisosira pseudonana* showed only a slight decrease of their C∶N ratios, a slight increase in red fluorescence and very few modifications in the expression of genes encoding for carbonic anhydrase or RUBISCO [16], suggesting that some diatom species may well adapt to acidification. These laboratory experiments already reveal that several physiological parameters are influenced by medium acidity, and that the response level may well depend on the species and/or be compensated for if the species has sufficient time to adapt.

Diatoms are found in many acidic or very basic freshwaters [17]. The species richness and their distribution across a large range of pH values make diatoms a good ecological indicator for monitoring environmental changes. For example, sediment-preserved diatom assemblages are used to reconstruct paleooceanographic events [18] and estimate freshwater pH values [19], [20]. Beside the active discussions about the number of pH specialists or generalists diatom species [21], [22], pH reconstruction data also rely on a good understanding of the phenotypic plasticity of individual diatom species, and therefore on a better understanding of the impact of local/global conditions on morphometric traits.

To our knowledge, no study has so far addressed the effects of external pH changes on the biomineralization process at the single cell and cell population levels. Using the marine diatom *Thalassiosira weissflogii*, we report that external pH (pHe) variations have various impacts on physiology, *e.g.*, growth rate and intracellular pH homeostasis, and on silicon biomineralization, *e.g.*, intracellular silicic acid and biogenic silica concentrations. We also develop several new approaches to show that pHe directly affects valve morphodynamics and valve pattern.

## Methods

### Cell culture

The diatom strain *Thalassiosira weissflogii* (Grun.) Fryxell and Hasle was obtained from the Provasoli-Guillard National Center for Culture of Marine Phytoplankton (CCMP-1051). Axenic cells were cultured in batch at low cell density (from 2×10^4^ to 3×10^5^ cells mL^−1^) in an artificial sea water medium (for details about the medium preparation see [23]), under a 12∶12 hr light-dark cycle with a light intensity of *ca.* 100 μEinstein m^−2^ s^−1^ (*ca.* 7,000 Lux), and at 19°C. After sterilization, the artificial sea water medium was completed by adding 175 µM silicic acid (a non-limiting concentration), dipotassium hydrogen orthophosphate and vitamins. The pH of the medium was adjusted to the expected value, using sterile 0.2 M HCl or 0.2 M NaOH, prior to and after sterilization. To measure the different parameters, including the kinetics of valve formation, *T. weissflogii* cells were first acclimated to the appropriate medium for 3 to 6 days, to ensure that the cells were in exponential growth phase. For each experiment, duplicated growth rate measurements were performed from independent cultures, and all conditions were cultured in parallel. Algal cell density was determined by flow cytometry counting.

### Intracellular pH value measurements

In order to measure cytosolic pH in diatoms, the cell-permeant form of the ratiometric H^+^-indicator BCECF (2′,7′bis(2carboxyethyl)5(and6)carboxyfluorescein) was used. *In vitro* calibration of BCECF-free acid (Life Technologies) was performed by loading 5 µM BCECF in a buffer containing approximate cytosolic concentrations of major ions (100 mM KCl, 30 mM NaCl, 500 mM mannitol, 25 mM MES, 25 mM HEPES; conductance = 11.07 mS cm^−1^) (Figure S1 *A*). The ratio of fluorescence emission at 535±30 nm was measured with sequential excitation at λ_1_ = 485±25 nm and λ_2_ = 436±10 nm. Images were acquired using a 100× PL APO 100×/1.40-0.70 Oil CS objective on a DM IRB microscope (Leica) coupled to a Cool Snap HQ CCD camera (Photometrics). To ensure correct adjustment along the z-axis, nanometric particles (PS-Speck™ Microscope Point Source, Life technology) were highly diluted and images were then collected at different Z-positions (+/−1 µm). *In situ* calibration was then performed to confirm that the cytosolic pH indicator responded to changes in intracellular pH in the same manner as for *in vitro* calibration. Cells loaded with BCECF-AM (Life Technologies) were exposed to the buffer mentioned above (except that [KCl] was 200 mM and conductance 18.18 mS cm^−1^). To allow for the complete equilibration of extracellular and intracellular H^+^ concentrations, 10 µM nigericin, an H^+^-K^+^ exchanger, and 10 µM monensin, an Na^+^-H^+^ exchanger, were also added. This procedure allows for relating the 485/395 background-corrected excitation ratios to pH values [24]. Different rows of cells were treated at external pH values ranging from 6.4 to 8.5 (Figure S1 *B*), and background fluorescence was determined in a row of unloaded cells. The average ratio of the central 50% of the cells (7 different planes were averaged, equivalent to 6 µm in depth) was used for calibration (Figure S1 *B*). The region of interest used to calculate the ratio corresponded to the whole cell. Background fluorescence was subtracted for each excitation.

To measure intracellular pH values, cells were first loaded with 10–12 µM BCECF-AM for 20 min, resulting in a final cytosolic concentration that yielded similar fluorescence intensity to the intensity obtained with the dye alone. To remove the unloaded dye, cells were rinsed twice with the culture medium, and resuspended in fresh medium. Again, for each cell the average fluorescence ratio of the center of the cell (6 µm) was considered. Again, the region of interest (ROI) used to calculate the ratio corresponded to the whole cell, and background fluorescence was subtracted for each excitation. Furthermore, for about half of the analyzed cells we made measurements in a ROI that corresponded to about 1/5^th^ of total cell area, and at a distance from any visible chloroplast. We did not find any important difference (*<*18%) in the fluorescence ratios between small regions and whole cells; this suggests that our measurements correspond to cytoplasmic signals (not shown).

### Determination of the biogenic and intracellular silicon pools

The biogenic silica content per cell (BSi quota) and the intracellular silicon content per cell (Sii quota) were determined using a procedure modified from a previous report [25]. Briefly, samples from cells in the exponential phase were first centrifuged (10 min, 1,500 rpm, 19°C) in Falcon tubes. The supernatant was discarded and the pellet was resuspended in 1 mL MilliQ H_2_0. Then, cells were lysed by treatment at 95°C for 10 min. After cooling, tubes were centrifuged again (10 min, 1,500 rpm, 19°C) and supernatants were used to estimate the intracellular silicon content per cell. Pellets were rinsed and washed twice with 1 mL MilliQ H_2_O before starting alkaline hydrolysis of biogenic silica with NaOH (0.2 M) for 45 min at 95°C. Hydrolyzates were then neutralized with HCl (1 M) and stabilized with Tris-HCl (10 mM final concentration, pH = 7.0). After centrifugation, the supernatants were used to measure biogenic silica contents per cell. All silicic acid concentrations were determined using the molybdenum-blue method.

### Microfluidics experiment setup, data acquisition and analyses

At the beginning of the light period, cells were concentrated by gentle centrifugation (1,200 rpm, 5 min, 19°C) and re-suspended in a fresh medium containing 1 µM HCK-123. At that concentration HCK-123 accumulation in the cytosol is very limited. However, we should keep in mind that in diatoms Lysotracker results depend on species, dye and concentrations [26], [27]. Then the cells were introduced by means of an external sample loop, using Medium Pressure Injection Valves (Upchurch Scientific®), into a rectangular (0.05 mm×1.00 mm) capillary device (VitroCom) connected via a NanoPort (Upchurch Scientific®) to PHD 2000 syringe pumps (Harvard Apparatus, USA). After cell sedimentation on the inner surface of the capillary, the pump was run at a slow rate of 1 µL hr^−1^, corresponding to a dilution rate of 2.2 s^−1^. Such a dilution rate allowed for constant renewal of the medium but generated some motility (see cell tracking below and Methods S1). Cells were imaged on a single plane every 5 to 7 min using the HC PL APO 10×/0.40 CS objective, whose calculated depth of field is about 8 µm. That way, most of the fluorescent signal of a single cell on a single plane (the width of our *T. weissflogii* cells was about 10 µm; Table S1) was inside the depth of field. In-between each acquisition of both bright-field and green fluorescence (HCK-123 signal), cells were illuminated under white collimated LED light (Thorlabs) set to *ca*. 100 μEinstein m^−2^ s^−1^ using neutral density filters. The capillary device, the entire microscope and the culture media were maintained at 19°C using a cell imaging box (Life Imaging Services). The total duration of the experiment was 8 to 10 hours, corresponding to a maximum of *ca.* 8 seconds of illumination in the green channel. Image analyses were performed using combined software, including Metamorph software 7.5 (Molecular Devices) and ImageJ 1.44 d.

### Cell tracking and image analysis

The automated detection and tracking of individual cells and the morphometric analyses were performed using specific shell-scripts and C modules written within the MegaWave2 environment (http://megawave.cmla.ens-cachan.fr/) (Methods S1). The time-course data at the 6 different pH values, from 6.2 to 8.5, correspond to a total of 31 independent experiments. To obtain the time-course data, we only considered the cells for which values were available at least 45 minutes before HCK-123 fluorescence reached the first maximum (F*_1_* see below) and whose *r* values for the fit of fluorescence as a function of time (*k*) were above 0.95.

### Frustule purification and morphometric measurements

Exponentially growing cells were harvested by centrifugation at low speed, and washed several times with distilled water. Organic material was first oxidized by potassium permanganate (final concentration 3%) with an excess of H_2_SO_4_, and then eliminated with 16% HNO_3_ (v/v) and 48% H_2_SO_4_ (2∶1, v/v) for 1 min. The suspension was neutralized by adding Tris-HCl buffer (1 M, pH 8.0) and then washed several times. A drop of the cleaned material was placed on a carbon-coated 200 mesh Nickel grid and observed with a Philips Tecnai 12 electron microscope. Among the traits measured (see Table S1) some were obtained using ImageJ software and the others were measured with newly developed semi-automatic approaches based on MATLAB software. Step-by-step image analysis is explained in Methods S1 and summarized in Figure S2.

### Statistical analyses

All statistical analyses were performed using XLSTAT. One-way ANOVAs followed by Tukey Honestly Significant Difference (HSD) tests (*p*<0.05) were used to evaluate the effect of environmental pH on the different parameters we studied. To estimate the differences in Sii and BSi quotas as a function of pHe, we used Mann-Whitney's test for non-parametric variables. For better visualizing data dispersion, most of the Figures are represented as boxplots. For each set of data, boxplots show the smallest observation, the lower quartile, the median, the upper quartile and the largest observations. In addition, mean values are represented by a cross in each box. To estimate the linear dependence between variables, we used Pearson correlation coefficient, and F-tests.

## Results

### Impact of the environmental pH on cell growth

To understand the potential impacts of the external pH (pHe) on diatom physiology, we used the centric species *Thalassiosira weissflogii* (Figure S3), a ubiquitous coastal marine diatom found in marine habitats [28]. We first determined growth rates in an enriched artificial sea water medium [26], and over a range of 2 pH units (pHe 6.4 to 8.5). In all cases the pH of the medium was controlled by organic buffers (Tris and MES, 17 mM and 3 mM, respectively) which ensured that the pH of the medium did not change significantly over a period of about 8 days, corresponding to the exponential growth phase. Such a broad range of pH values is wider than the one predicted by the different acidification scenarios [29], but is compatible with observed seasonal fluctuations and daily variations in coastal ecosystems [30].

**Figure 1:**
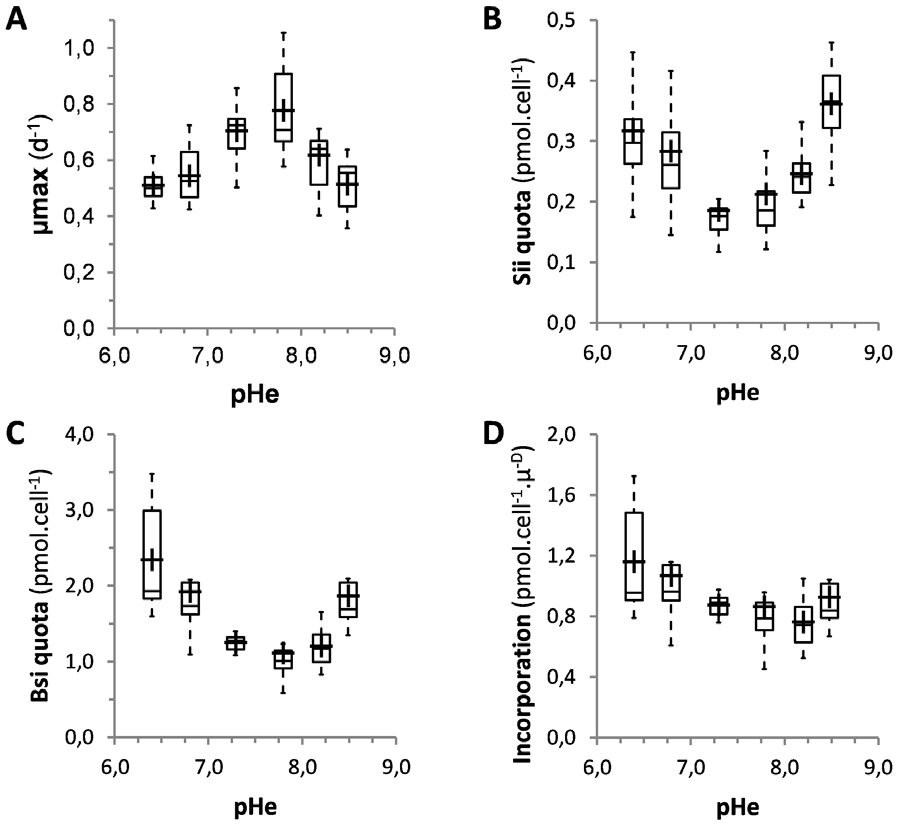
Influence of the external pH on cell growth and silicon metabolism in *T. weissflogii*. (**A**) Variations of the maximal growth rate (μmax) at different environmental pH values. Boxplots correspond to 5 to 11 independent experiments. The highest growth rate at pH = 7.8 was confirmed in individual experiments, but was only significant as compared to pH = 6.4, 6.8 and 8.5 (*p*≤0.007). (**B**) Intracellular silicic acid (Sii) content per cell was determined from cells grown in artificial sea medium adjusted to different pH values. (**C**) Frustule-bound BSi content per cell. Boxpolts in **B** and **C** correspond to 4 to 10 independent experiments, with duplicate measurements for each experiment. (**D**) Boxplot illustrating the silicon incorporation rate, *i.e.*, the rate of frustule formation (pmol cell^−1^ μ^−D^) as a function of environmental pH. For explanations about boxplot representation, see METHODS.

For this diatom the fastest division rate of *ca.* 32±7 hours (*n* = 11 experiments, with some experiments performed in duplicate) was found for a pHe value of 7.8, slightly lower than the average surface ocean pH. Analyses of growth rates calculated from cells in exponential phase revealed that acidification or basification of the medium led to decreased growth rates (Figure 1*A*). These results give indications on the capability of *T. weissflogii* to get acclimated to short-term variations in environmental pH, from circumneutral to slightly basic.

### The environmental pH affects Si metabolism

We investigated the effect of pHe on Si(OH)_4_ accumulation in *T. weissflogii*. On average, we found that growth in acidic or basic conditions led to a gradual increase of intracellular silicic acid contents (Sii quota) (Figure 1*B*). Compared to the Sii quota found at pHe = 7.8, we found that Sii quota increased 1.5- or 1.7-fold in the most acidic (pHe = 6.4) or the most basic (pHe = 8.5) conditions, respectively (Figure 1*B*) (*p*<0.001).

To test whether changes in pHe would also influence the Si biomineralization process, we measured the biogenic silica content per cell (BSi quota; pmol cell^−1^). Compared to the apparent optimal growth pH value of 7.8, the BSi quota was 2.1- or 1.7-fold higher at pHe = 6.4 and pHe = 8.5, respectively (*p*<0.0003). Moreover, the overall variations of BSi and Sii quotas displayed similar trends over the range of pH values tested (compare Figures 1*B and 1C*).

Another estimation of the influence of pHe on Si metabolism was obtained by calculating silicon incorporation which is directly related to the rate of frustule formation, calculated as BSi content per cell divided by specific growth rate. We found that Si(OH)_4_ incorporation decreased from pHe = 6.4 to 8.2, and then increased from 8.2 to 8.5 (Figure 1*D*). These data reveal that the longer the cell spends in cell division, the more silicon is incorporated into the frustule, and the opposite occurs under high growth rates. However, even if our data demonstrate that the external pH affects silicification, the lowest incorporation rate was observed at pHe = 8.2, but not at pHe = 7.8, the highest growth rate pHe value. This suggests that other factors than cell division duration are involved in the regulation of Si metabolism.

### External acidification leads to lower intracellular pH values

To better understand the influence of the external pH on cell physiology, we developed for the first time experiments to assess the ability of *T. weissflogii* to control its internal pH (pHi). Direct measurements of cytosolic pH (pHi) of living *T. weissflogii* cells were performed using the fluorescent H^+^-indicator BCECF. BCECF has been used to measure intracellular pH in a large number of organisms including several unicellular algae [31], [32], [33]. Although BCECF-AM sequestration in organelles (*e.g.*, mitochondria, nuclei, pollen tube organelles) has sometimes been observed, the signal from the bulk cytoplasm was generally present too (see discussion in [34]).

**Figure 2:**
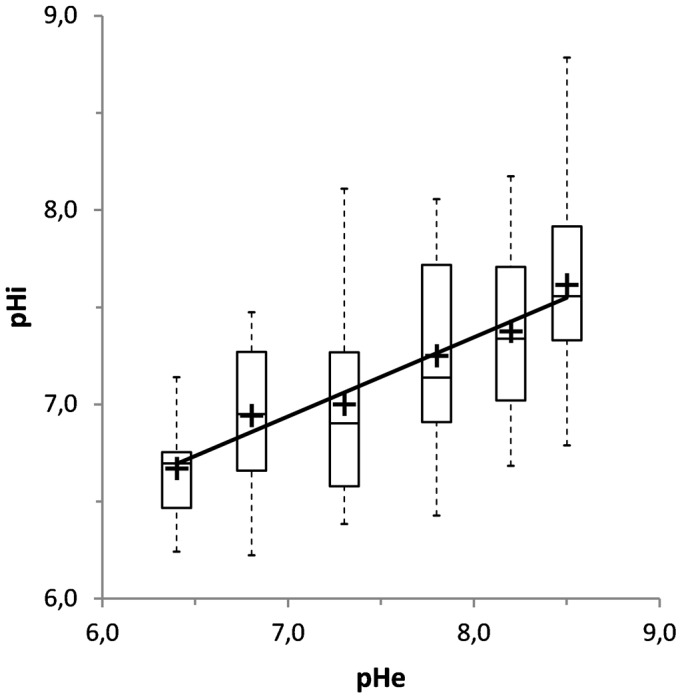
Influence of external pH on intracellular pH. *Thalassiosira weissflogii* cells were loaded with BCECF-AM at different external pH values (pHe). Ratiometric emission (with excitation at 485 and 436 nm) was used to calculate intracellular pH values (pHi). The relation between pHe and pHi measurements was fitted to a linear regression (*r^2^* = 0.306; Fisher's, *p*<0.0001), and corresponds to 20≤*n*≤30 measurements.

We used *in vivo* calibration and signals corresponding to whole cells to measure intracellular pH (see METHODS). We found that at the optimal growth rate pHe value of 7.8, the pHi value was 7.35 (*n* = 30). This intracellular pH value is close to the ones found in other algae [31], [35]. We also found a linear relationship between the internal pH and the range of external pH values (Figure 2). Thus, a pHe variation of 2.1 units led to a pHi decrease of 0.94 unit (*p*<0.0001), which is equivalent to an increase of the intracellular proton concentration by *ca.* 8.8 fold. Even if our results reveal the capability of *T. weissflogii* to counterbalance variations in external pH, they also demonstrate that acidification induces a significant intracellular acidosis.

### Measurement of valve formation dynamics

**Figure 3:**
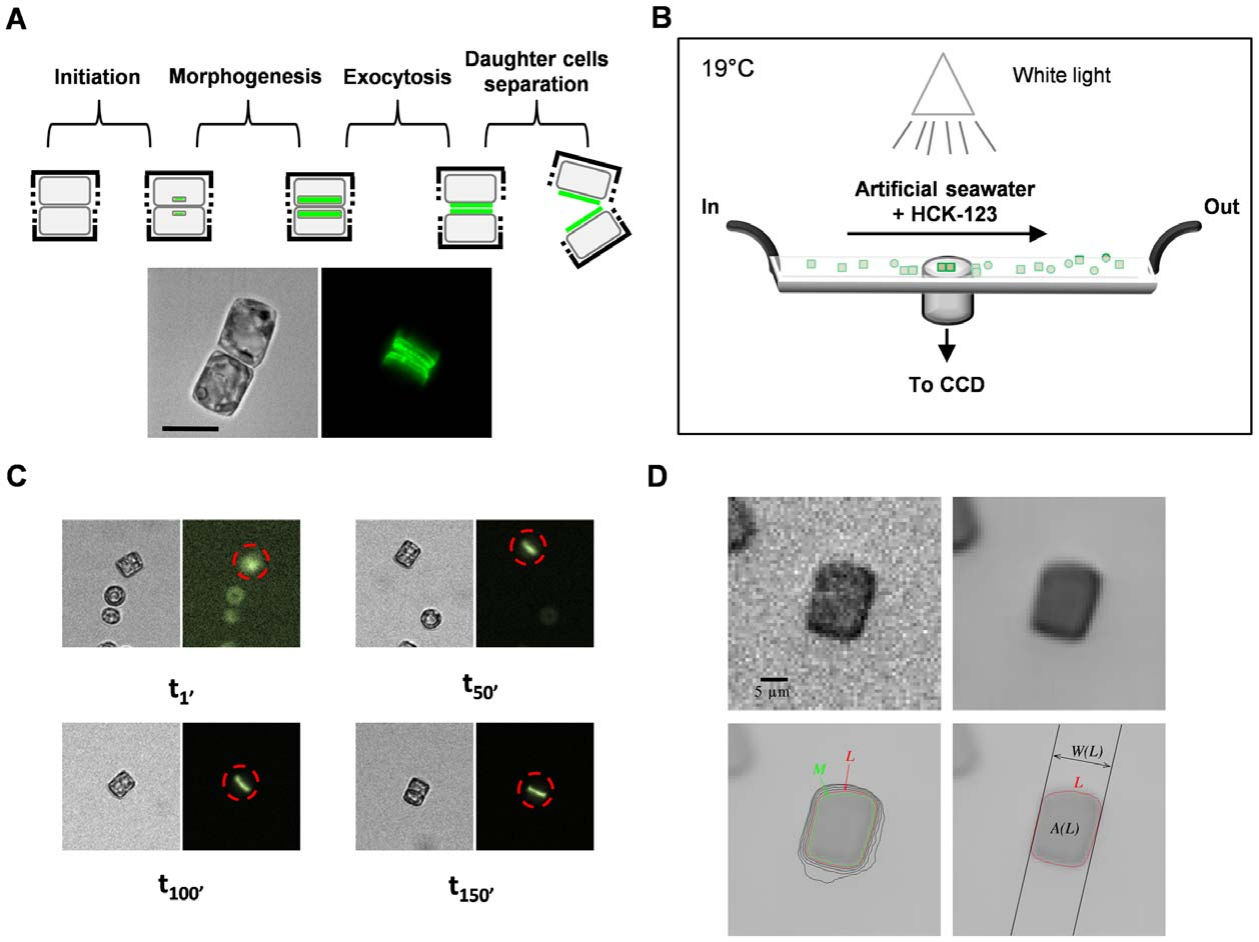
Measurement of valve formation dynamics at the single-cell level. (**A**) The valve formation process can be separated into several phases: an initiation phase, followed by valve formation and morphogenesis, then by valve exocytosis and finally by daughter-cell separation. The two images correspond to the DIC (digital image correlation) and Z-projections of HCK-123 fluorescence (*green*). In green we can visualize newly-synthesized valves labeled with the silica-associated dye (HCK-123). Scale bar (*black*): 10 µm. (**B**) Schematic representation of the microfluidic device used to record valve formation. During valve formation, light intensity, temperature and renewal of the medium were controlled. (**C**) To quantify HCK-123 fluorescence in individual cells, we developed new software for cell-tracking and local background estimation (Methods S1). Images at 4 different times are presented. Apparent cell motility is caused by the liquid flow. (**D**) To precisely quantify fluorescence in each cell we developed a new shape extraction method. The original image was denoised with the TV-means algorithm, leading to a much cleaner image. Then, among the level lines (iso-intensity curves) of the image enclosing the known center of the cell, we considered that the cell boundary was the line with the sharpest contrast (*L*). This enabled us to compute cell area *A(L)* as the area of the region enclosed by *L*), and its width *W(L)*, defined as the minimum width of a band containing *L*.

To better understand the effects of varying external pH on valve formation (Figure 3*A*), we developed a number of new approaches to measure the kinetics of frustule formation in real time.

Over the last years, a number of fluorescent dyes have been used to label the formation of neo-synthesized frustule in diatoms [26], [36], [37]. These permeant probes, which generally consist of a fluorophore linked to a weak base, typically concentrate in acidic organelles as a result of their protonation. To visualize valve formation in live conditions, we favored the use of a dye named HCK-123 (see [26] and Figure 3*A*). As additional controls, we tested that the fluorescence of HCK-123 incorporated into silica material depended on its concentration in the growth medium (Methods S1 and Figure S4), and that in solution its fluorescence properties did not change over a 3.0 to 8.0 pH range (Figure S5); such range is compatible with the estimated pH (*ca.* pH = 5) inside Silica Deposition Vesicles (SDVs) [38]. We also checked that HCK-123 fluorescence did not vary according to the buffers used (Methods S1), and calibrated HCK-fluorescence intensity as a function of its concentration in our capillary device (Figure S6). Altogether our data suggest that HCK-123 fluorescence is independent of pH and supports its use as a quantitative reporter to monitor its accumulation into acidic compartments in living cells.

Exponentially growing *T. weissflogii* cells were introduced into a simple micro-fluidic device, with controlled temperature and light intensity. Moreover, in order not to affect the rate of valve synthesis that could induce modifications unrelated to environmental buffering, we performed measurements on un-immobilized cells and under constant medium renewal (Figure 3*B*). This latter condition induced apparent cell motility. Therefore, to record the time course of HCK-123 incorporation into individual cells, we also developed a number of specific shell-scripts and C-modules. Altogether our tools allowed us to perform cell tracking, cellular fluorescence quantification over the local background, and semi-automatic measurements of cell dimensions (Figures 3*C–D* and Methods S1). In addition, to quantitatively evaluate the evolution of HCK-123 concentrations, we performed a series of complementary experiments using a serial dilution of HCK-123 and quantified the signal using the same procedure as the one used for the cells (Methods S1). We obtained a calibration curve of HCK-123 fluorescence in our microfluidic device (Figure S6). That latter calibration, combined with cell dimension (*i.e.*, cell width *W(L)* and cell area *A(L)*) calculations, allowed us to calculate HCK-123 concentrations *per* cell biovolume, (*i.e.*, the volume of a single dividing cell) (Methods S1).

**Figure 4:**
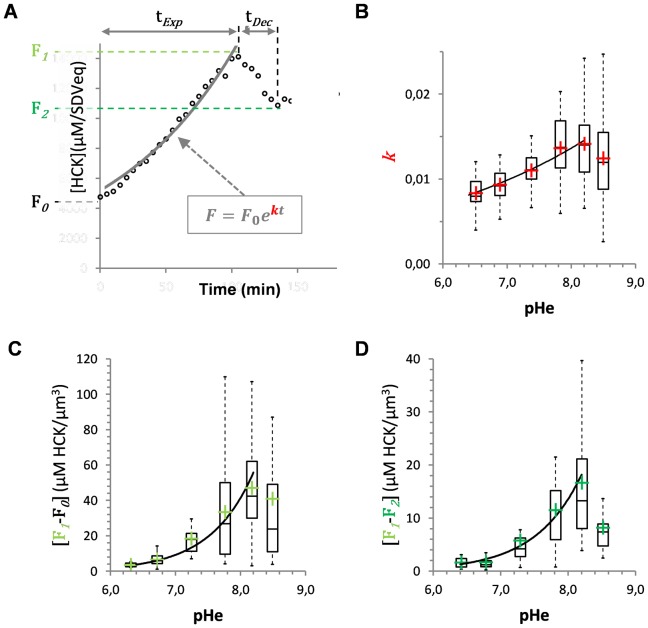
Influence of pHe on the kinetics of valve formation. (**A**) The initial phase of valve formation is composed of two phases: an exponential evolution of the HCK-123 signal (*t*_Exp_) that ends at a maximum named F*_1_*, followed by a decay phase that ends at the minimum (*t*_Dec_), named F*_2_*. During the first phase we fitted the data to an exponential and determined the slope (*k*) and the coordinates on the y-axis (F*_0_*). (**B**) Influence of extracellular pH on *k*. In the 6.4–8.2 pH range, data were fitted to an exponential (y = 1.1e-3.e^0.32×^; *r^2^* = 0.98). (**C**) Variation of HCK-123 concentrations as a function of external pH during the exponential (F*_1_*-F*_0_*) phase. In the 6.4–8.2 pH range, data were fitted to an exponential (y = 1e-4.e^1.50×^; *r^2^* = 0.98). (**D**) Variation of HCK-123 concentrations during the decay (F*_1_*-F*_2_*) phase. In the 6.4–8.2 pH range, data were fitted to an exponential (y = 5e-5.e^1.47×^; *r^2^* = 0.95). Data are from 7 to 92 single-cell measurements.

These image analyses performed over a period of *ca.* 8 hours and from cells grown at pHe = 7.8 (the measured optimal-growth-rate pH value) provided the first time-resolved data on the kinetics of valve formation (Movie S1). Our results reveal that the initial phases of valve formation can be separated into two periods: a first period, during which HCK-123 accumulates as a function of time, and that is best fitted to an exponential (*t*_Exp_; Figure 4*A*), and a second period that corresponds to a decrease (*t*_Dec_; Figure 4*A*) in total fluorescence intensity. This second period was found in approximately 75% of the cells, revealing the existence of cell-to-cell variability. During the second period (*t*_Dec_), assimilated to a maturation phase, the lower HCK-123 signal suggests that a proportion of “non-incorporated” dye was released. Such a decrease of the HCK-123 concentration can be ascribed to a decrease of the intra-SDV pH and/or to a decrease of overall SDV volume (see Discussion). We calculated the overall duration of these two early periods (*t*_Exp_ and *t*_Dec_) from 6 independent experiments. The results were 90±39 min (*n* = 54) and 47±20 min (*n* = 29), respectively (Figure S7). Such duration of the valve formation process is compatible with previous estimates from population studies [36], [39], [40], [41]. During the experiments we managed to visualize the complete process for a few cells, from the early events up to daughter cell separation (Movie S2). These data reveal that diatoms can regulate conditions inside SDVs during valve formation, as evidenced by the evolution of HCK-123 fluorescence. In addition, the existence of several phases also supports that silicon biomineralization could be a non-uniform process.

### Influence of the environmental pH on the dynamics of valve formation

Starting from the recording of several hundreds of cells, we obtained time course data for individual *T. weissflogii* cells grown at pH = 6.4, 6.8, 7.3, 7.8, 8.2 and 8.5. Recalling the existence of cell-to-cell variability, we found that the pHe changed the overall duration of t_Exp_ and of t_Dec_ but within a limited range of <1.5-fold (Figure S7). To further test the influence of the environmental pH on the dynamics of valve formation, we calculated the rate of dye incorporation (*i.e.*, total HCK-123 concentrations as a function of time) during the exponential phase. We found that the highest speed (*k*) was found at pHe = 8.2 (Figure 4*B*), and that more basic or more acidic pH values slowed down the speed of the morphogenesis process. These results clearly demonstrate that the external pH has a direct impact on the kinetics of SDV expansion and/or silica polymerization.

We also estimated [HCK-123] variations per SDV-equivalent (Methods S1) over the exponential (F*_1_*-F*_0_*) and decay (F*_1_*-F*_2_*) phases, as a function of pHe. We found that the deduced dependence of [HCK-123] on the extracellular proton concentration was similar in both the exponential and the decay phases (Figures 4*C–D*). We calculated that a variation of 1.8 pHe units (8.2 minus 6.4) induced 13.8- and 10.6-fold variations of the fluorophore concentration for the exponential and the decay phases, respectively. These data demonstrate that the dynamics and the extent of valve formation vary according to the extracellular pH, and also to intracellular pH to some extent.

### Impact of the environmental pH on valve pattern

**Figure 5:**
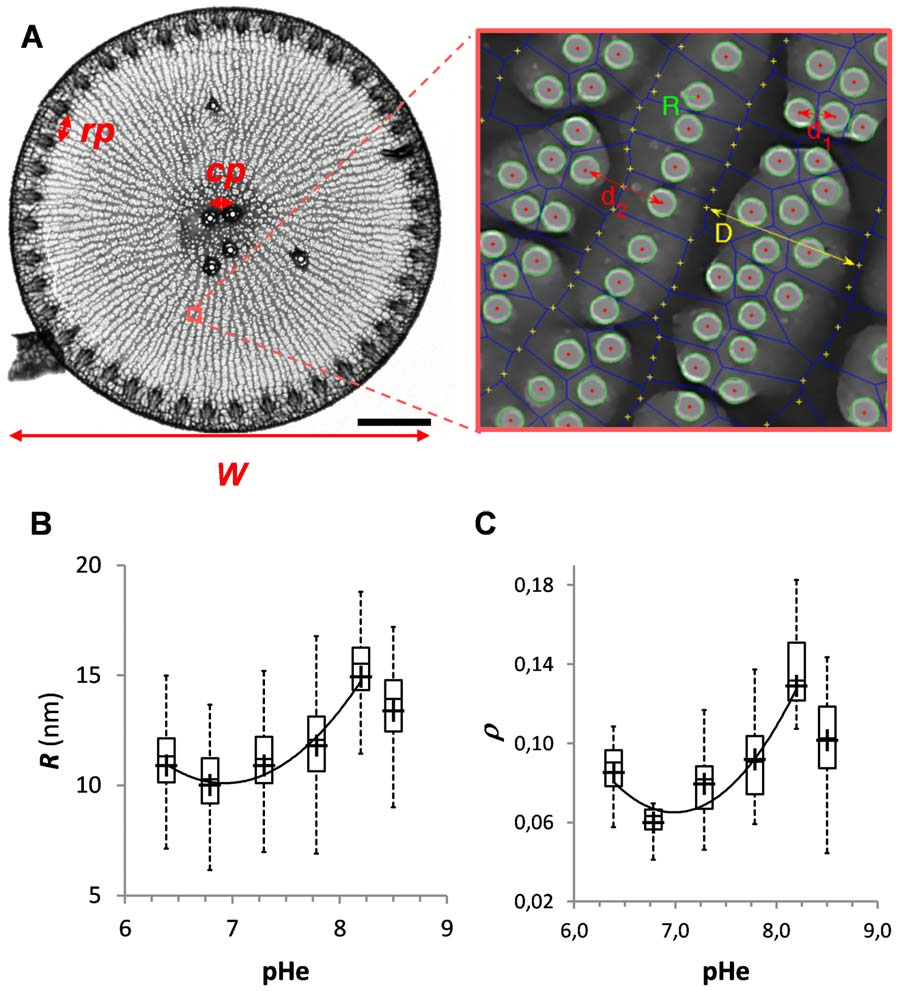
Influence of the environmental pH on valve morphometry. (**A**) TEM image of a valve showing the different traits measured: valve width (*W*), number (*N*) and distance (*cp*) between the fultoportulae present in the central region, and distance between rimoportulae (*rp*). Scale bar: 5 µm. The right panel corresponds to a higher magnification (64,000×) showing an array of pores and the computed Voronoi diagram (*dark blue lines*) (Methods S1). We measured pore radius (*R*), the average distance between two adjacent pores (*d1*), the width of groups of pores, also known as semi-continuous cribrum (*D*), and the width of radial ribs (*d2*). (**B**) Boxplot showing variations in pore radiuses (*R*) (971≤*n*≤3551) as a function of external pH (pHe). (**C**) Boxplot showing the influence of pHe on overall valve porosity (*r*) (7≤*n*≤21).

To further test the implications of external pH variations on valve formation, we analyzed the morphometry of valves purified from cells grown at the different pHe values. We used a combination of image processing techniques, from manual estimates to semi-automated ones, to precisely quantify variations in valve morphology; from overall morphology (valve length, number of fultoportulae, distance between adjacent rimoportulae …) to the architecture of fine structures (width of semi-continuous cibra, pore diameter …). Altogether the valves were characterized by 9 different morphometric traits (Figures 5*A*).

While most of the traits did not present any significant variation upon pHe modifications (Table S1 and Figure S8), nano-sized pore dimension presented a maximum at pHe = 8.2 and then decreased upon acidification or basification of the medium (Figure 5*B*). From high magnification (64,000×) images, we also estimated overall valve porosity. We found that compared to valves purified from cells grown at pHe = 8.2, the silica network porosity decreased 2.2-fold (*i.e.*, 0.058/0.128) at pHe = 6.8 or 1.3-fold (*i.e.*, 0.100/0.128) at pHe = 8.5 (Figure 5*C*). Interestingly, the variations in nano-pore diameter and valve porosity seemed to follow the same trend-line as the variations observed for the kinetics of valve formation as a function of the pHe; this suggests a link between morphogenesis speed and some morphometric traits.

## Discussion

In this study, we examine the influence of the external/environmental pH on several physiological parameters, valve morphogenesis and theca pattern in the ubiquitous coastal marine diatom *Thalassiosiria weissflogii* [28]. This centric (cell with radial symmetry) diatom species has been the subject of a large number of studies that have allowed for important developments in diatom physiology, biochemistry and ecology. The species belongs to the order *Thalassiosirales*, a lineage composed by a large diversity of species distributed across a number of marine but also freshwater habitats [42]. Molecular studies estimate *Thalassiosirales* to be at least 100 million years old [43], making the *Thalassiosirales* excellent models for studying diatom evolution and adaptation. Additionally the genus studied here, *Thalassiosira* Cleve, displays morphology features that are characteristic of fossil and modern taxa [44]. Therefore understanding the impact of the environmental pH on *T. weissflogii* will help to understand not only the biology of this species, but also the phenotypic variability of morphological features that are common to a large number of diatom species.

### External pH influences cell physiology and Si metabolism

There is a large consensus that the increasing amount of CO_2_ released in the atmosphere has already changed a variety of physical and chemical properties. Such changes in *p*CO_2_/ocean pH values have already been shown to have consequences on marine biogenic calcification [45], [46], and on organisms that biomineralize silicon [47]. Some studies show that acidification does not lead to any difference in specific growth rates in *T. pseudonana*, *T. weissflogii* [16], [48], [49], or *Nitzschia* spp. [50]. Other experiments show that increased *p*CO_2_ could lead to increased growth rates in *Pseudo-nitzschia fraudulenta* [15], *Pseudo-nitzschia multiseries* [14], *Skeletonema costatum* [48] or *Chaetoceros muelleri* [51]. Most of these studies compare the physiology of diatoms generally grown under low *p*CO_2_ (100–200 ppm), modern atmospheric *p*C0_2_ (350–400 ppm) or high *p*CO_2_ (730–770 ppm). This range corresponds to pH variations from about 8.5 down to 7.8. Our laboratory experiments aimed to investigate the capability of *T. weissflogii* to cope with broad variations in environmental pH, over a range of 2 pH units (pHe 6.4 to 8.5). Such pH range is broader than the one predicted by the different acidification scenarios [29], but is compatible with observed seasonal fluctuations and daily variations in coastal ecosystems [30].

We first found that from the optimal pH value of 7.8, both more acidic or basic external pH values led to decreased growth rates in *T. weissflogii*. Conversely, changes in pHe also resulted in modifications of the Si(OH)_4_ metabolism. In fact, both the intracellular Si content and the frustule-bound silicon content quotas increased upon moderate acidification and at the most basic pHe. These results agree well with a previous study by Milligan *et al.* (2004) who found a small (*ca.* 1.25-fold) but significant increase in BSi at a high pH value (8.4) [49], and further demonstrate the influence of acidic growth conditions on *T. weissflogii* silicification. The negative correlation between mineralization and growth rate is consistent with previous data on pH, nutrient limitation or other stressors in diatoms [52], [53], [54], [55], [56]. A previous interpretation proposed that the longer the cells spent in the G2 and M phases (the time of valve formation), the more silicon was incorporated into the neo-synthesized silica material [52]. While our results support that interpretation, they also suggest that the Si incorporation rate does not simply reflect cell cycle duration. Indeed, we found that the trend line of the evolution of silicon incorporation as a function of pHe differed from the growth rate trend line (compare Figures 1*A and 1D*), suggesting that carbon-concentrating mechanisms [49], or other biological processes such as membrane transport processes, metabolic functions, or maintenance of cellular pH homeostasis, also contribute to regulating silicon metabolism, and are sensitive to external pH changes.

### External acidification induces intracellular pH modifications

As mentioned before, external pH modifications also affect the metabolic state of the cell due to regulation of the acid-base balance. Modifications of the intracellular pH induced by variations in environmental pH have been measured in several phytoplankton lineages, including several Chlorophyceae, Streptophytae or Haptophyta [32], [33], [57], [58], [59], [60], [61], [62], but to our knowledge had never been addressed in diatoms.

Using the pH-sensitive probe BCECF and single-cell measurements, we found that the intracellular pH (pHi) of *T. weissflogii* significantly varied upon modification of the external pH. We estimated that the pHi decreased from 7.6 at an external pH value of 8.5, down to 6.7 at an external pH value of 6.4. Although experiments with long-term adaptation at different external pH values and transient external pH modifications will be needed to better characterize the buffering capacity of diatoms, our data already reveal that external acidification leads to intracellular acidosis.

In living cells, several processes (cell proliferation, differentiation, and cell cycle progression) and biological molecules (ionization state of metabolites, protein folding, polar head groups of membrane phospholipids …) are pH-dependent (for a review see [63]). In diatoms, a first study that used specific inhibitors of a class of proton transporter (*e.g.*, V-ATPases) showed that disturbances of the activity of these transporters could impede frustule formation in the model diatom *Phaeodactylum tricornutum*, and alter theca pattern [28]. Another important issue, specifically related to the biomineralization process, is the regulation of organelle pH [64]. Indeed, in eukaryotes most organelles have their own specific pH, and modifications of the endogenous acid-base balance could also lead to pH modifications in these cellular organelles [65], [66].

In diatoms, the vesicle within which silicon biomineralization occurs, namely the silica deposition vesicle (SDV), is an acidic compartment [38]. Tight regulation of the physicochemical conditions inside SDVs is therefore considered as important for a proper regulation of silica polycondensation [67]. Moreover, alterations of the acid-base balance inside SDVs can also modify the interactions between the mineral phase and the organic compounds known to be directly involved in silica precipitation, but also among these organic compounds themselves. All these data strengthens the importance of acid-base balance regulation in diatom metabolism. Our data also raise a question about the potential implications of acidification not only on the maintenance of intracellular pH, but also on the regulation of SDV pH.

### Environmental pH influences the extent of the biomineralization process

We combined our kinetics data on valve formation (*i.e.*, the variations of HCK-123 concentrations *per* SDV-equivalent and as a function of time) to modeling to interpret the impact of external pH on SDV expansion.

**Figure 6:**
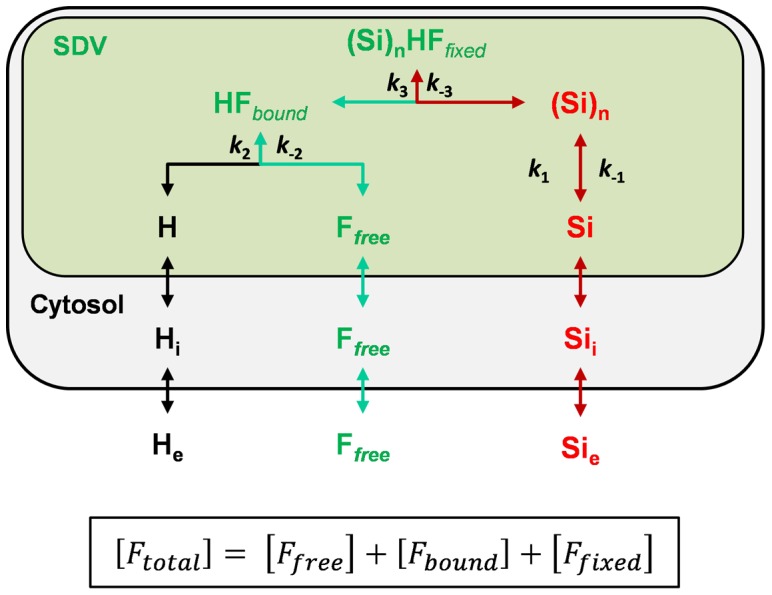
Model used to estimate the equilibrium inside Silica Deposition Vesicles (SDVs). We considered that the fluorescent dye F*_free_* freely diffused inside the diatom cells. Inside SDVs, the dye is protonated and accumulates (HF*_bound_*) as a function of SDV pH. Upon SDV expansion, which follows a non-linear kinetics, HF*_bound_* concentrations increased exponentially. At the same time an important fraction of the dye got entrapped inside the newly-formed silica material ((Si)_n_ HF*_fixed_*). Therefore, [HCK] inside SDVs corresponds to the sum of the free, bound and fixed fractions.

We first derived an analytical model restricted to some of the main components [H^+^], [Si] and [HCK-123], involved in valve formation (Figure 6). According to this simple model, total fluorescence corresponds to the sum of three different fractions:
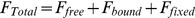
(1)where [F*_free_*] is the [HCK-123] that freely diffuses and accumulates at very low concentrations inside cells and SDVs (we estimated that the fraction inside cells was negligible); [F*_bound_*] is the HCK-123 fraction “immobilized” inside SDVs as a result of protonation (the SDV compartment is acidic); and [F*_fixed_*] is the [HCK-123] entrapped inside the silica material (Methods S1). As we previously showed that purified frustules remain fluorescent [32], we assume that the fraction of the fluorophore that is already incorporated into the biogenic silica fraction cannot dissociate (*i.e.*, dissolution is not considered in our model). This hypothesis fits well with the current knowledge on diatom biomineralization which does not evidence any *in vivo* dissolution process. Our simplified model proved helpful to interpret the initial phases of valve formation. During the first phase (*t*_Exp_; Figure 4*A*), total fluorescence intensity increases exponentially as a function of time. As we suspect HCK concentrations to be quickly equilibrated, the increase in [F*_Total_*] is interpreted as the result of the polycondensation process itself (increase in [F*_fixed_*]), and concurrently of the increase in SDV volume. During the second phase (*t*_Dec_; Figure 4*A*), considered as a maturation phase, [F*_Total_*] decreases. Following our initial “no dissolution” assumption, such decreases can be accounted for by a decrease in SDV volume and/or a change in SDV pH (more basic pH values in our case); both processes lead to a decrease of [F*_bound_*].

For these two initial phases, we estimated that a drop in the external pH value from 8.2 to 6.4, which corresponds to an intracellular pH decrease of about 0.7 unit (between pHi = 7.38 and 6.67; *p*<0.0003), led to a change in [HCK-123] by about one order of magnitude (13.9-fold for *t*_Exp_, the first phase of valve formation, and 10.9-fold for *t*_Dec_, the second period of valve formation). As the same drop in external pH value from 8.2 to 6.4 led to a higher biogenic silica quota we can infer that modifying the external pH, and thereby the internal pH balance, induces a modification of SDV pH and/or volume. As mentioned above, as similar changes in [HCK] were found for both the exponential and the decay phases, modifications of the external pH possibly led to a change in intra-SDV pH by an estimated 1 pH unit. Whatever the exact value, our results support the idea that external pH influences the regulation of the acid-base balance inside SDVs.

Finally, assuming that [HCK-123] is a reliable indicator of the events that occur inside SDVs, our data indicate that SDVs probably have their own intrinsic pH regulatory system and that the buffering capacity of the cytoplasm could indirectly affect SDV pH homeostasis. This interesting hypothesis, which has to be confirmed, should be further investigated.

### Influences of environmental pH on the morphogenesis process and valve pattern

In diatoms, variations of valve morphology are well documented and used as markers of changes in aquatic ecosystems [68]. The availability of major and minor nutrients or the presence of pollutants is known to induce valve morphology modifications or abnormalities. For example, changes in salinity alter a number of morphological traits of the silica frustule in both marine and fresh-water diatoms [55], [56], [69], [70]. Flexibility in morphometrics and reduced nanometric pore dimension were reported at low salinity levels for the marine diatom species *T. weissflogii* and *T. punctigera* [55], [56], [69].

By analyzing a number of morphological traits from purified frustules of *T. weissflogii* cells grown at different external pH values valve (Figures 5*A* and Figure S2), we found that the pHe induced variations in the size of the nanometric pores of the semi-continuous cibra located on the inner side of the valve (Figures 5*B*). Overall valve porosity was also significantly lower in cells grown at a pHe value of 6.8 compared to 8.2. Interestingly, statistical analyses also showed that the differences in pore diameter (*p*<0.0001) and valve porosity (*p* = 0.004) were already significant when comparing pHe = 7.8 cells to pHe = 8.2 cells. As the estimated numbers of pores found on the semi-continuous cibra were roughly similar (not shown), the difference in porosity results from a difference in pore extension rather than a difference in the initiation of the pore-forming process. Interestingly, our results also suggest that modifications of valve porosity cannot be explained by the sole variations in biogenic silicon concentrations (compare Figures 5*B–C* to Figure 1*C*).

In fact, our modeling and kinetics data evidence that pattern formation is also regulated by SDV expansion speed (*i.e., k*). At the *k* optimum pH (pHe = 8.2) (see Figure 4*B*) quicker SDV expansion led to the formation of larger pores, and probably of a more developed branching network on the outside of the valve. Upon acidification or basification, biomineralization speed decreases, allowing for more silica to condense and formation of smaller pores. In these conditions we also found that the silicon incorporation rate was higher (Figure 1*D*). The observed changes in *T. weissflogii* silica valve morphology can have several implications on cell physiology. Indeed, without important modifications of the overall valve morphology, variations of the valve diffusive layer are expected to modify nutrient supply and uptake capability in diatoms. Reduced inner pore size can decrease the diffusion coefficient as a result of confinement. Alternatively, changes in pores shape and dimension can increase solvent and solute advection (for a review see [4]). Changes in frustule morphology are also expected to affect its mechanical properties [71]. Finally, modifying frustule porosity according to external pH might find applications to finely control the properties of diatom thecae. Altogether these data strengthen the need for further investigations on the consequences of modifications of the silica nanostructure on outward and inward transport through the frustule.

## Conclusion

Our data reveal that (i) a disturbance of environmental pH has pleiotropic effects on *T. weissflogii* physiology, including impacts on growth rate, silicon metabolism and intracellular pH homeostasis, (ii) the regulation of the intracellular acid-base balance plays a central role in the regulation of silicon metabolism in diatoms. The putative impacts of the environmental pH are therefore expected to vary among different diatoms species according to their species-specific intrinsic buffering capacity, and adaptation capability. Due to its general importance, intracellular pH regulation should also be investigated in studies on carbon acquisition, transport capability or intravesicular pH regulation, for example.

We also present for the first time evidence that disturbances of biomineralization dynamics partially explain the modifications of theca morphology induced by variations in external pH. Our results highlight the importance of coupling single cell analyses with morphometric studies to understand pattern formation in diatoms. More precisely, the time course and the speed of vesicle expansion and co-occurring silicon polycondensation are key factors to understand silica morphogenesis in these algae.

## Supporting Information

Table S1 **Influence of the external pH on valves morphometry.** The eight morphometric traits measured on purified valves from *T. weissflogii* cells grown at the different pHs are: the average cell width and the number (N) of central fultoportulae, the minimum distance (l) between two adjacent central fultoportulae, the minimum distance (L) between two adjacent rimoportulae, the radius (R) of the pores, the distance (*d_1_*) between two adjacent pores, the width of semi-continuous cribra (D), the width of radial ribs (*d_2_*), and the valve porosity.
(DOCX)

Figure S1 **Calibration curves used to measure the intracellular pH.** (**A**) Background corrected 485/436 ratios of BCECF. The *in vitro* calibration corresponds to BCECF-free acid (5 µM) in a wide range of pH buffer values. Mean and SD correspond to three independent experiments and from 8 to 30 measures. (**B**) *In situ* calibration of BCECF-AM (5 µM) loaded cells after cells had been treated with ionophores to equilibrate the intracellular and extracellular H^+^-concentrations. The data correspond to two independent experiments, with 11≤*n*≤21 measures.
(PDF)

Figure S2 **Step by step image analyses for the extraction of several valve morphometric traits.** (**A**) Original TEM image. The scale bar corresponds to 100 nm. (**B**) The same image after noise reduction. (**C**) Image after binarization. (**D**) Determination of the circular regions which correspond to the valve pores (*in green*), directly allowed us to determine the pore radius (*r*) and the valve porosity (*r*). It also allows to compute the corresponding Voronoi diagram (*blue lines*). (**E**) Histogram of distances between two neighbors (*d*) in the Voronoi diagram. (**F**) Determination of points defining the fingers (*yellow crosses*). (**G**) Determination of the second Voronoi diagram. (**H**) Measurement of distance *D*, across fingers. (**I**) Histogram of the distance *D*.
(PDF)

Figure S3 **Scanning electron micrographs of the centric diatom** ***Thalassiosira weissflogii*** **.** (**A**) View of the valve exterior showing the network on loculate areola. Note the presence of central fultoportulae, and of rimoportulae. (**B**) Semicontinuous cribra are present on the valve face. The interior opening of rimoportulae and the fultoportulae is visible. The scale bar corresponds to 5 µm.
(PDF)

Figure S4 **Boxplots showing the fluorescence intensity of cells labeled with different concentrations of HCK-123.** *T. weissflogii* cells grown for 24 hours in the presence of HCK-123 were analyzed by flow cytometry, with a total of 3,800 to 5,800 analyzed cells. These results support the idea that HCK-123 is quantitative incorporated into the newly synthesized silica frustules.
(PDF)

Figure S5 **Fluorescence properties of the Lysotracker HCK-123.** Influence of the pH on the maximum emission of 1 µM HCK-123 in either 100 mM potassium hydrogen phosphate buffer or in 20 mM phosphate/citrate buffer. The intensity was normalized to the value obtained at pH = 7.0.
(PDF)

Figure S6 **Calibration of the fluorescence intensity as a concentration of HCK-123.** *In situ* determination of the fluorescence signal for 9 different concentrations (between 0 and 62.5 µM) of HCK-123. The method used was the same method as the one used to determine the signal inside *T. weissflogii* cells. The data that correspond to 3 independent experiments from 36 to 150 measurements were fitted to a linear curve.
(PDF)

Figure S7 **Duration of the initial phases of valve formation.** The duration of the two initial periods of valve formation was determined from cells grown at different pHs. (**A**) Length of the exponential phase (*t_Exp_*). (**B**) Length of the decay phase (*t_Dec_*). The data that correspond to 3 to 9 independent experiments were extracted from the recording of 8 to 92 individual cell kinetics.
(PDF)

Figure S8 **Influence of the environmental pH on valve morphology.** We used TEM images at resolution level from the nanometer to the micron-scale to test the impact of the environmental pH on morphometric traits of the valve. (**A**) The width (*W*) of the valve (10≤*n*≤21). (**B**) The number (*N*) of central fultoportulae per cell (10≤*n*≤22). (**C**) The minimum distance between fultoportulae present in the central region (*cp*) cell (35≤*n*≤82). (**D**) Distance between two adjacent rimoportulae (*rp*) (28≤*n*≤128). (**E**) The distance (*d_1_*) between two adjacent pores (54,842≤*n*≤78,374). (**F**) The width of branching ribs (*d_2_*) (54,842≤*n*≤78,374). (**G**) The distance between the ribs (*D*) (6,378≤*n*≤15,038). The representations from ***A*** to ***D*** correspond to boxplots, and in ***E*** to **G** only the mean and the standard deviation are presented.
(PDF)

Movie S1 **Dynamics of frustule formation in** ***T. weissflogii*** **.** This real-time movie shows the evolution of the fluorescence of the dye used to follow valve formation in diatoms. The entire movie corresponds to 31 images acquired at 3.33 mHz, and the quantification of the HCK-123 is presented in Figure 4*A*. The left panel corresponds to the quantification and the right panel to the images of the HCK-123 fluorescence.
(AVI)

Movie S2 **Valves formation and separation of daughter cells.** These movies which correspond to 97 images acquired at 3.33 mHz, reveal the process of cell separation. The left panel corresponds to the DIC images and the right panel to the images of the HCK-123 fluorescence.
(AVI)

Methods S1 **The supplemental methods contains: assessment of the use of HCK-123 as a reporter for valve formation, image analyses, morphometric analyses, modeling, and additional references.**
(DOCX)
