# Supplementary material for: Multiparametric Analyses Reveal the pH-Dependence of Silicon Biomineralization in Diatoms
Source: PLoS One. 2012 Oct 29;7(10):e46722. doi: 10.1371/journal.pone.0046722 (PMC3483172; doi:10.1371/journal.pone.0046722)
Supplement: Table S1 — Influence of the external pH on valves morphometry. The eight morphometric traits measured on purified valves from T. weissflogii cells grown at the different pHs are: the average cell width and the number (N) of central fultoportulae, the minimum distance (l) between two adjacent central fultoportulae, the minimum distance (L) between two adjacent rimoportulae, the radius (R) of the pores, the distance (d1) between two adjacent pores, the width of semi-continuous cribra (D), the width of radial ribs (d2), and the valve porosity. (DOCX) [file pone.0046722.s001.docx]

**Legend of Supplementary Table S1**

**Table S1. Influence of the external pH on valves morphometry.**

^a^ The average cell width and the number (N) of central fultoportulae / cell were determined from 10 to 21 valves.

^b^ The minimum distance (l) between two adjacent central fultoportulae corresponds to 35 to 82 independent measurements.

^c^ The minimum distance (L) between two adjacent rimoportulae corresponds to 28 to 128 independent measurements.

^d^ The radius (R) of the pores corresponds to 971 to 3551 measurements.

^e^ The distance (*d_1_*) between two adjacent pores corresponds to 5596 to 20570 measurements.

^f^ The width of semi-continuous cribra (D) corresponds to 7 to 21 measurements.

^g^ The width of radial ribs (*d_2_*) corresponds to 5596 to 20570 measurements.

^h^ The valve porosity corresponds to 7 to 21 measurements.
