# Supplementary material for: Multiparametric Analyses Reveal the pH-Dependence of Silicon Biomineralization in Diatoms
Source: PLoS One. 2012 Oct 29;7(10):e46722. doi: 10.1371/journal.pone.0046722 (PMC3483172; doi:10.1371/journal.pone.0046722)
Supplement: Figure S1 — Calibration curves used to measure the intracellular pH. (A) Background corrected 485/436 ratios of BCECF. The in vitro calibration corresponds to BCECF-free acid (5 µM) in a wide range of pH buffer values. Mean and SD correspond to three independent experiments and from 8 to 30 measures. (B) In situ calibration of BCECF-AM (5 µM) loaded cells after cells had been treated with ionophores to equilibrate the intracellular and extracellular H+-concentrations. The data correspond to two independent experiments, with 11≤n≤21 measures. (PDF) [file pone.0046722.s002.pdf]

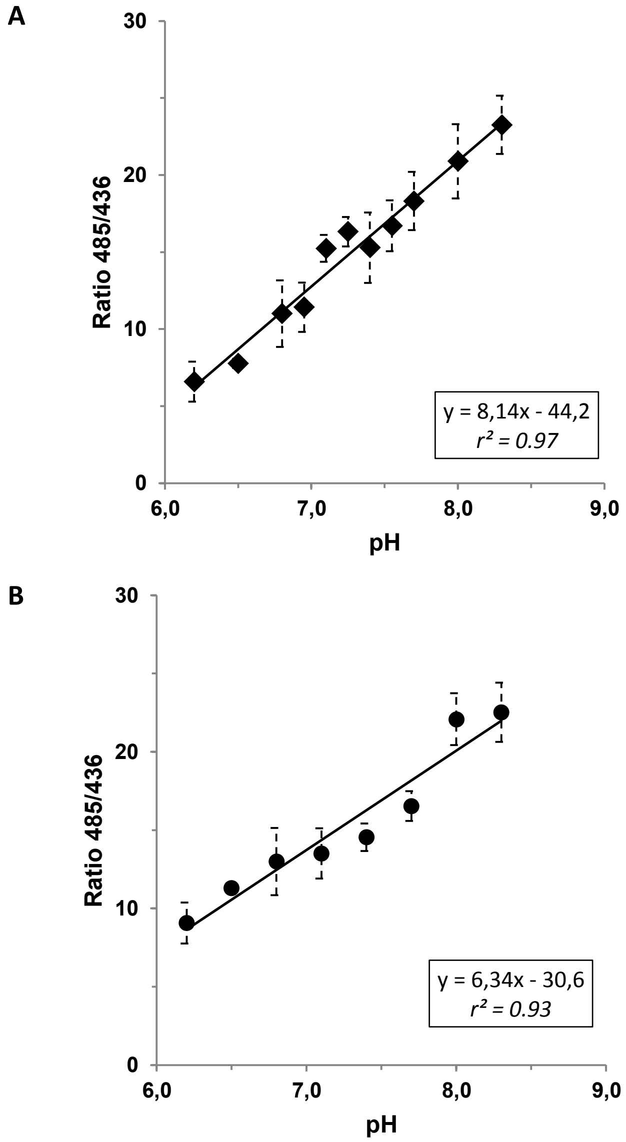

Figure S1. Calibration curves used to measure the intracellular pH.

(**A**) Background corrected 485/436 ratios of BCECF. The *in vitro* calibration corresponds to BCECF-free acid (5 µM) in a wide range of pH buffer values. Mean and SD correspond to three independent experiments and from 8 to 30 measures. (**B**) *In situ* calibration of BCECF-AM (5 µM) loaded cells after cells had been treated with ionophores to equilibrate the intracellular and extracellular H^+-concentrations. The data correspond to two independent experiments, with $11 \leq n \leq 21$ measures.
