# Supplementary material for: Multiparametric Analyses Reveal the pH-Dependence of Silicon Biomineralization in Diatoms
Source: PLoS One. 2012 Oct 29;7(10):e46722. doi: 10.1371/journal.pone.0046722 (PMC3483172; doi:10.1371/journal.pone.0046722)
Supplement: Figure S2 — Step by step image analyses for the extraction of several valve morphometric traits. (A) Original TEM image. The scale bar corresponds to 100 nm. (B) The same image after noise reduction. (C) Image after binarization. (D) Determination of the circular regions which correspond to the valve pores (in green), directly allowed us to determine the pore radius (r) and the valve porosity (r). It also allows to compute the corresponding Voronoi diagram (blue lines). (E) Histogram of distances between two neighbors (d) in the Voronoi diagram. (F) Determination of points defining the fingers (yellow crosses). (G) Determination of the second Voronoi diagram. (H) Measurement of distance D, across fingers. (I) Histogram of the distance D. (PDF) [file pone.0046722.s003.pdf]

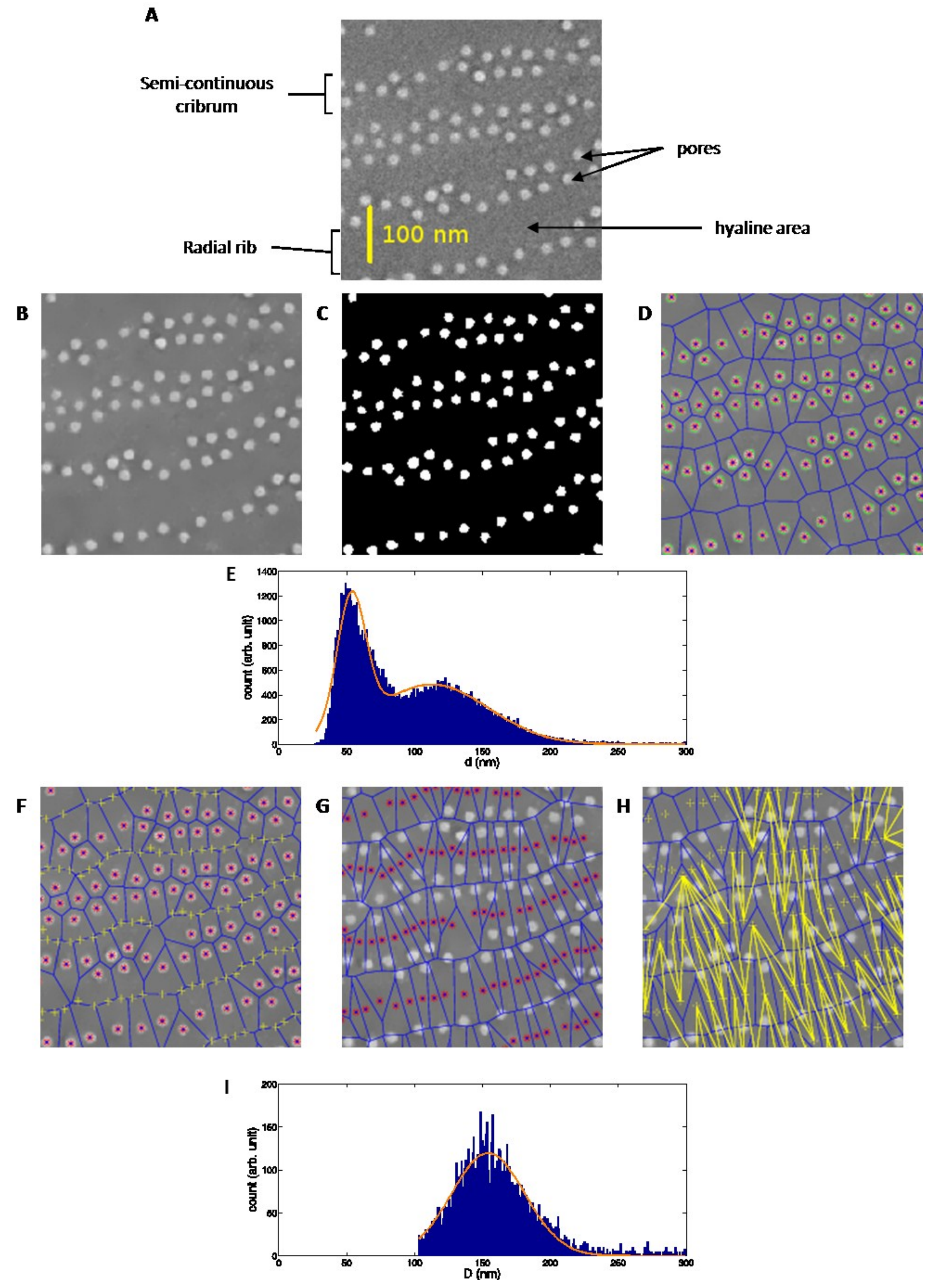

Figure S2. Step by step image analyses for the extraction of several valve morphometric traits.
(**A**) Original TEM image. The scale bar corresponds to 100 nm. (**B**) The same image after noise reduction. (**C**) Image after binarization. (**D**) Determination of the circular regions which correspond to

the valve pores (*in green*), directly allowed us to determine the pore radius (*r*) and the valve porosity (*r*). It also allows to compute the corresponding Voronoi diagram *(blue lines*). (**E**) Histogram of distances between two neighbors (*d*) in the Voronoi diagram. (**F**) Determination of points defining the fingers (*yellow crosses*). (**G**) Determination of the second Voronoi diagram. (**H**) Measurement of distance *D*, across fingers. (**I**) Histogram of the distance *D*.
