# Supplementary material for: Multiparametric Analyses Reveal the pH-Dependence of Silicon Biomineralization in Diatoms
Source: PLoS One. 2012 Oct 29;7(10):e46722. doi: 10.1371/journal.pone.0046722 (PMC3483172; doi:10.1371/journal.pone.0046722)
Supplement: Figure S3 — Scanning electron micrographs of the centric diatom Thalassiosira weissflogii . (A) View of the valve exterior showing the network on loculate areola. Note the presence of central fultoportulae, and of rimoportulae. (B) Semicontinuous cribra are present on the valve face. The interior opening of rimoportulae and the fultoportulae is visible. The scale bar corresponds to 5 µm. (PDF) [file pone.0046722.s004.pdf]

A

B

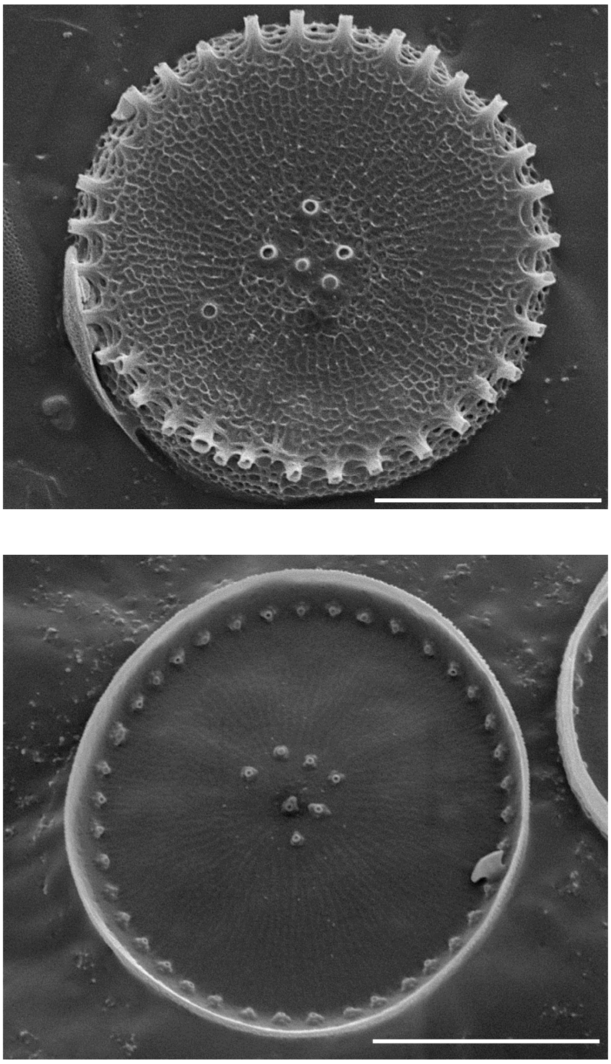

Figure S3. Scanning electron micrographs of the centric diatom *Thalassiosira weissflogii*.
(**A**) View of the valve exterior showing the network on loculate areola. Note the presence of central fultoportulae, and of rimoportulae. (**B**) Semicontinuous cribra are present on the valve face. The interior opening of rimoportulae and the fultoportulae is visible. The scale bar corresponds to 5 µm.
