# Supplementary material for: Multiparametric Analyses Reveal the pH-Dependence of Silicon Biomineralization in Diatoms
Source: PLoS One. 2012 Oct 29;7(10):e46722. doi: 10.1371/journal.pone.0046722 (PMC3483172; doi:10.1371/journal.pone.0046722)
Supplement: Figure S4 — Boxplots showing the fluorescence intensity of cells labeled with different concentrations of HCK-123. T. weissflogii cells grown for 24 hours in the presence of HCK-123 were analyzed by flow cytometry, with a total of 3,800 to 5,800 analyzed cells. These results support the idea that HCK-123 is quantitative incorporated into the newly synthesized silica frustules. (PDF) [file pone.0046722.s005.pdf]

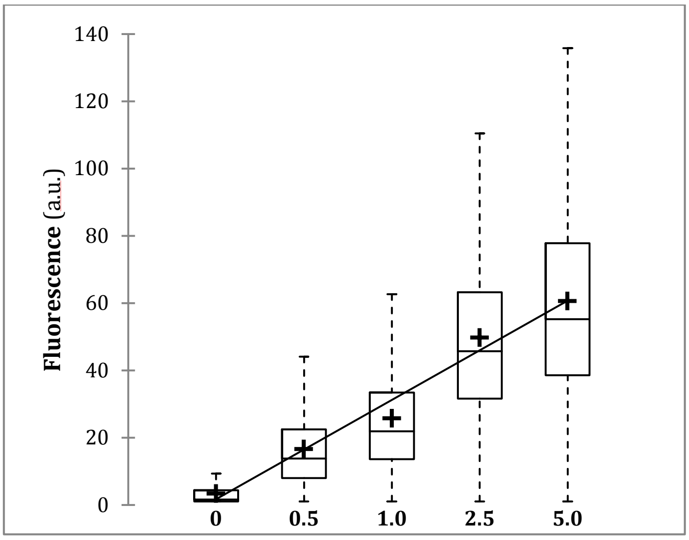

[HCK-123] (µM)

Figure S4. Boxplots showing the fluorescence intensity of cells labeled with different concentrations of HCK-123.

T. weissflogii cells grown for 24 hours in the presence of HCK-123 were analyzed by flow cytometry, with a total of 3,800 to 5,800 analyzed cells. These results support the idea that HCK-123 is quantitative incorporated into the newly synthesized silica frustules.
