# Supplementary material for: Multiparametric Analyses Reveal the pH-Dependence of Silicon Biomineralization in Diatoms
Source: PLoS One. 2012 Oct 29;7(10):e46722. doi: 10.1371/journal.pone.0046722 (PMC3483172; doi:10.1371/journal.pone.0046722)
Supplement: Figure S5 — Fluorescence properties of the Lysotracker HCK-123. Influence of the pH on the maximum emission of 1 µM HCK-123 in either 100 mM potassium hydrogen phosphate buffer or in 20 mM phosphate/citrate buffer. The intensity was normalized to the value obtained at pH = 7.0. (PDF) [file pone.0046722.s006.pdf]

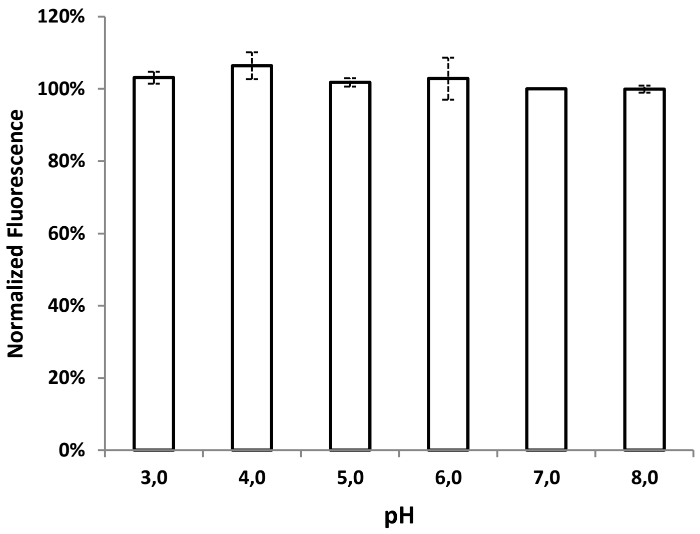

Figure S5. Fluorescence properties of the Lysotracker HCK-123.
Influence of the pH on the maximum emission of 1 µM HCK-123 in either 100 mM potassium hydrogen phosphate buffer or in 20 mM phosphate/citrate buffer. The intensity was normalized to the value obtained at pH=7.0.
