# Supplementary material for: Multiparametric Analyses Reveal the pH-Dependence of Silicon Biomineralization in Diatoms
Source: PLoS One. 2012 Oct 29;7(10):e46722. doi: 10.1371/journal.pone.0046722 (PMC3483172; doi:10.1371/journal.pone.0046722)
Supplement: Figure S6 — Calibration of the fluorescence intensity as a concentration of HCK-123. In situ determination of the fluorescence signal for 9 different concentrations (between 0 and 62.5 µM) of HCK-123. The method used was the same method as the one used to determine the signal inside T. weissflogii cells. The data that correspond to 3 independent experiments from 36 to 150 measurements were fitted to a linear curve. (PDF) [file pone.0046722.s007.pdf]

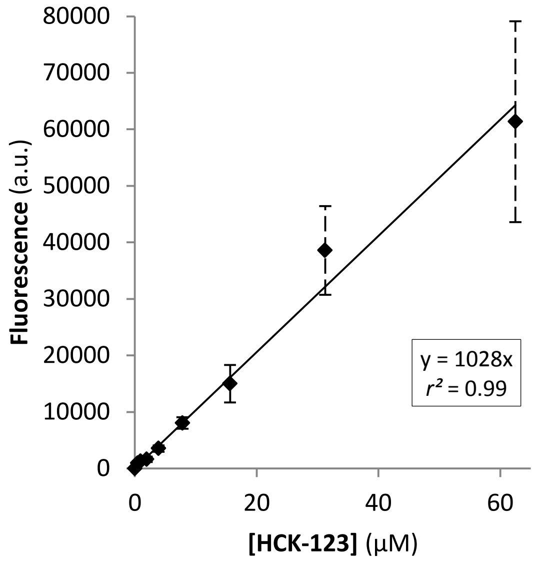

Figure S6. Calibration of the fluorescence intensity as a concentration of HCK-123.
In situ determination of the fluorescence signal for 9 different concentrations (between 0 and 62.5 µM) of HCK-123. The method used was the same method as the one used to determine the signal inside *T. weissflogii* cells. The data that correspond to 3 independent experiments from 36 to 150 measurements were fitted to a linear curve.
