# Supplementary material for: Multiparametric Analyses Reveal the pH-Dependence of Silicon Biomineralization in Diatoms
Source: PLoS One. 2012 Oct 29;7(10):e46722. doi: 10.1371/journal.pone.0046722 (PMC3483172; doi:10.1371/journal.pone.0046722)
Supplement: Figure S7 — Duration of the initial phases of valve formation. The duration of the two initial periods of valve formation was determined from cells grown at different pHs. (A) Length of the exponential phase (tExp). (B) Length of the decay phase (tDec). The data that correspond to 3 to 9 independent experiments were extracted from the recording of 8 to 92 individual cell kinetics. (PDF) [file pone.0046722.s008.pdf]

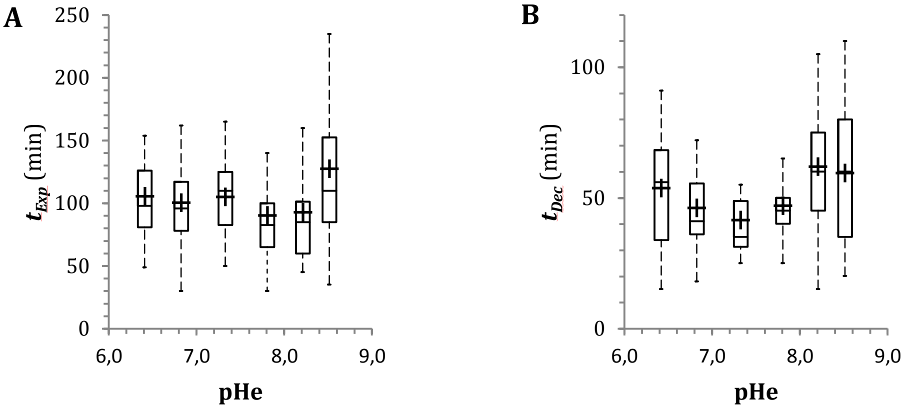

Figure S7. Duration of the initial phases of valve formation.

The duration of the two initial periods of valve formation was determined from cells grown at different pHs. (**A**) Length of the exponential phase (t_{Exp}). (**B**) Length of the decay phase (t_{Dec}). The data that correspond to 3 to 9 independent experiments were extracted from the recording of 8 to 92 individual cell kinetics.
