# Supplementary material for: Multiparametric Analyses Reveal the pH-Dependence of Silicon Biomineralization in Diatoms
Source: PLoS One. 2012 Oct 29;7(10):e46722. doi: 10.1371/journal.pone.0046722 (PMC3483172; doi:10.1371/journal.pone.0046722)
Supplement: Figure S8 — Influence of the environmental pH on valve morphology. We used TEM images at resolution level from the nanometer to the micron-scale to test the impact of the environmental pH on morphometric traits of the valve. (A) The width (W) of the valve (10≤n≤21). (B) The number (N) of central fultoportulae per cell (10≤n≤22). (C) The minimum distance between fultoportulae present in the central region (cp) cell (35≤n≤82). (D) Distance between two adjacent rimoportulae (rp) (28≤n≤128). (E) The distance (d1) between two adjacent pores (54,842≤n≤78,374). (F) The width of branching ribs (d2) (54,842≤n≤78,374). (G) The distance between the ribs (D) (6,378≤n≤15,038). The representations from A to D correspond to boxplots, and in E to G only the mean and the standard deviation are presented. (PDF) [file pone.0046722.s009.pdf]

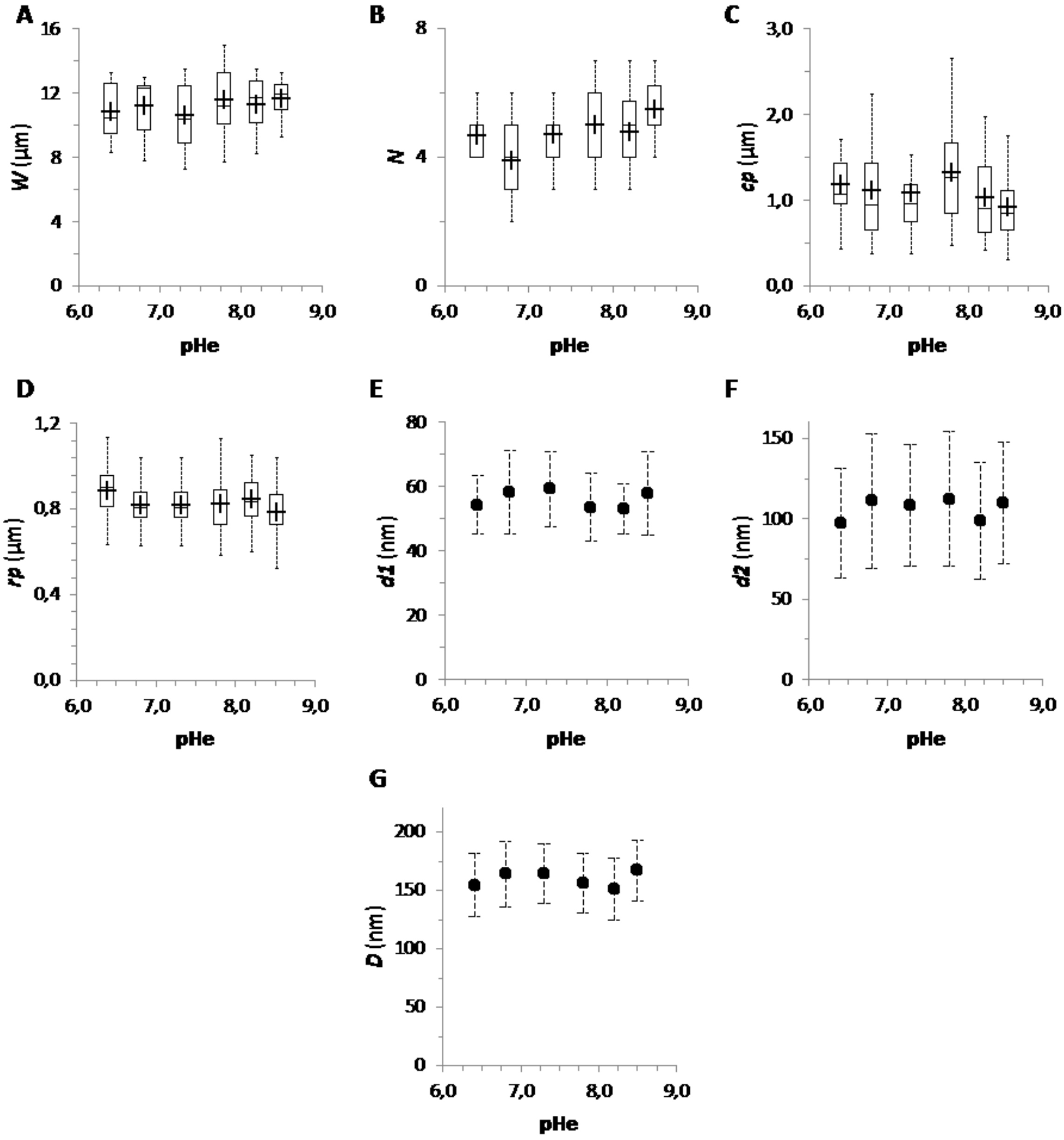

Figure S8. Influence of the environmental pH on valve morphology.

We used TEM images at resolution level from the nanometer to the micron-scale to test the impact of the environmental pH on morphometric traits of the valve. (**A**) The width (*W*) of the valve (10 ≤ *n* ≤ 21). (**B**) The number (*N*) of central fultoportulae per cell (10 ≤ *n* ≤ 22). (**C**) The minimum distance between fultoportulae present in the central region (*cp*) cell (35 ≤ *n* ≤ 82). (**D**) Distance between two adjacent rimoportulae (*rp*) (28 ≤ *n* ≤ 128). (**E**) The distance (d_1) between two adjacent pores (54,842 ≤ *n* ≤ 78,374). (**F**) The width of branching ribs (d_2) (54,842 ≤ *n* ≤ 78,374). (**G**) The distance between the ribs (*D*) (6,378 ≤ *n* ≤ 15,038). The representations from ***A*** to ***D*** correspond to boxplots, and in ***E*** to **G** only the mean and the standard deviation are presented.
