# Supplementary material for: Multiparametric Analyses Reveal the pH-Dependence of Silicon Biomineralization in Diatoms
Source: PLoS One. 2012 Oct 29;7(10):e46722. doi: 10.1371/journal.pone.0046722 (PMC3483172; doi:10.1371/journal.pone.0046722)
Supplement: Methods S1 — The supplemental methods contains: assessment of the use of HCK-123 as a reporter for valve formation, image analyses, morphometric analyses, modeling, and additional references. (DOCX) [file pone.0046722.s012.docx]

# Supplemental methods S1

## Assessment of the use of HCK-123 as a reporter for valve formation

The Lysosensor DND-160 or rhodamine derivatives [[1](#_ENREF_1)] have been used to estimate the accumulation of silicic acid [[2](#_ENREF_2)] and to follow the deposition of biogenic silica [[3](#_ENREF_3),[4](#_ENREF_4),[5](#_ENREF_5),[6](#_ENREF_6),[7](#_ENREF_7)]. It was shown that its incorporation into diatoms silica material is quantitative [[8](#_ENREF_8)]. These probes, which generally consist of a fluorophore linked to a weak base, are permeant to cells membrane and typically concentrate in acidic organelles. It is assumed, even if it has not been firmly established, that their mechanism of retention inside acidic compartment involves protonation. We have previously demonstrated that another Lysosensor HCK-123, is also a very useful to label newly synthesized silica materials in live diatoms [[9](#_ENREF_9),[10](#_ENREF_10)]. Here, we first confirmed that at the concentration used (1 µM) HCK-123 did not affect cell growth (not shown), and then performed a series of complementary experiments to test for its use as a quantitative reporter for silica formation in diatoms.

We measured the fluorescence of cells labeled with different concentrations of HCK-123. *T weissflogii* cells grown in the presence of 0.5 µM, 1 µM, 2.5 µM or 5 µM final HCK-123 concentrations were analyzed by flow cytometry after 24 hours. The flow cytometric analyses were carried out on a Cell Lab Quanta™ SC flow cytometer (Beckman Coulter, CA, USA). A 488-nm laser was used for excitation, and the green fluorescence was collected through a (525 ± 30)-nm band-pass filter. Data acquisition was done at a low flow-rate (*ca*. 5 µl.min^-1^) for 3 to 10 min depending on the concentration of the target population (which varies between 1 to 5.10^-5^ cell ml^-1^). Cytograms were analyzed using Cell Lab Quanta software for cell counts, and XLSTAT software (Addinsoft, France) for further analyses. The Figure S4 reveals a linear incorporation of HCK-123 into diatoms silica material. Indeed, without the 24 hours of incubation, which allows the synthesis of new valves, the HCK-123 accumulation within the cells was, in our conditions, negligible.

We then tested the stability of HCK-123 (Invitrogen) over a range of pH compatible with the estimated pH (*ca*. pH=5 [[11](#_ENREF_11)]) inside the Silica Deposition Vesicle (SDV). We found in a 20 mM potassium hydrogen phthalate buffer, no modification of both the excitation (λmax=475 nm) and emission (λmax=550 nm) spectrum over a range of pH from 3.0 to 7.0 (Figure S5), when measured at 23°C (Xenius XC, SAFAS Monaco). We also checked that other buffers such as Tris-HCl, citrate, sea water medium or the presence of up to 50 µM silicic acid do not modify the HCK-123 fluorescence properties (not shown). Altogether our data reveal that the fluorescence of HCK-123 is independent to the pH, and suggest that it’s a good reporter to monitor its accumulation into acidic compartments.

## Image analyses

### Cell Tracking

Cell tracking in our image sequences was relatively easy, because the cell density was small, so that in general the distance between neighboring cells remained large compared to the typical displacement of a cell between two successive images. This is why we used a very simple tracking method, based on thresholding and proximity.

First, we applied a fixed threshold to the images, and kept the connected components whose area was above a minimum size (10 pixels). We then considered the centers of mass of these regions as the potential cell centers. Then, given the known cell position *x_n_* in image *n* (the position in the first image being marked manually), we computed the position in image *n*+1 by taking, among all potential cell centers in image *n*+1, the one at position *y* leading to the smallest displacement *d*_1_ = |*y - x_n_*|. To control the robustness of the tracking, we provided to the supervisor the value of *d*_2_/*d*_1_, where *d*_2_ was the second smallest value of |*y* - *x_n_*| (that is, among other potential cell centers of image *n*+1). High values of *d*_2_/*d*_1_ mean that the nearest neighbor choice is robust, and thus indicate that the tracking is probably correct.

This method sometimes failed, in particular in the case of sudden cell motion or agglutinated cells. These special cases were processed manually, and in the end all the obtained cell trajectories were visually inspected.

An improvement of the method, that significantly reduced the manual processing (in particular in the early stage of the green fluorescence), was to consider the fluorescence images (instead of the Nomarsky images), and to restrict the nearest-neighbor search to the top 10 brightest connected components (the brightness being simply defined as the maximum intensity value in the considered connected component).

### Estimation of the signal intensity

Once the cell centers were found, we computed, for each cell position *x*, the associated intensity signal by integrating the image intensity *I* on a fixed disc centered in *x*, leading to the value:

$S=\sum_{y,|y-x|\leq r} \left( I\left( y \right)-\mu\right)$ (1)

where µ was an estimate of the background local mean value. To obtain a robust (but slightly biased) value for µ, we considered the minimum average intensity encountered around *x*, the average being taken on the same domain shape as the one used to compute *S*, that is, a disc with radius *r*. Precisely we computed:

$\mu=\min_{z,|z-x|\leq R} \frac{\sum_{y,|y-z|\leq r} I\left( y \right)}{\#\left\{ y,|y-z|\leq r \right\}}$ (2)

In practice *R* = 50 and *r* = 15 seemed to be appropriate. The underlying idea was that if other cells were present in the neighborhood of *x*, they would increase some average intensity values but would not influence too much the minimum average value. In other terms, our local background level estimate remains relevant as long as there exist at least one void (cell-free) disc region around *x*.

### Shape extraction

The shape extraction was performed in the following way (Figure 3*D*). First, the image (in practice a 61x61 image crop centered at the estimated cell center) was denoised with the TV-means algorithm [[12](#_ENREF_12)], that is a combination of Total Variation denoising [[13](#_ENREF_13)] and Non-Local means [[14](#_ENREF_14)]. Using 11x11 patches and a denoising level equals to σ = 8 seemed to be an appropriate choice. Second, the level lines of the denoised image were analyzed. Recall that given a digital image $I : \Omega\text{→}\mathbb{R}$ defined on a discrete domain$\Omega\subset\mathbb{Z}^{2}$, we can define its level lines as the boundaries of its upper level sets$\left( U_{\lambda} \right)_{\lambda\mathbb{\in R}}$, given by

$\forall\lambda\mathbb{\in R}, U_{\lambda}=\left\{ x\in\Omega, I(x)\geq\lambda\right\}$ (3)

The level lines of *I* are exactly the connected components of the boundaries of these level sets, and they naturally present a tree structure: a level line $M$ is a descendant of a level line *L* if $Int(M) \subset\mathrm{Int}\left( L \right)$, where $\mathrm{Int}\left( L \right)$ denotes the interior of $L$, that is, the bounded region enclosed by the Jordan curve $L$. This tree structure can be efficiently computed with the so-called Fast Level Set Transform [[15](#_ENREF_15)]. We defined the cell boundary by considering, among all level lines that enclosed the estimated (approximate) cell center, the one that had the largest contrast

$c\left( L \right)={max}_{M} (\text{}\left( L \right)-\text{}\left( M \right))$ (4)

where $\text{}\left( L \right)$ denotes the threshold used to define $L$, and the maximum is taken among all level lines $M$ such that $Int(M) \subset\mathrm{Int}\left( L \right)$ and $\mathrm{area}\left( \mathrm{Int}\left( L \right) \right)- area(Int\left( M \right))\leq s$, the positive number $s$ being a fixed area parameter (10 pixels in practice). To avoid potential small structures due do remaining noise, we restricted the analysis to level lines enclosing at least 100 pixels.

For each shape $L$ obtained with this process, we computed two associated measurements: the area $A(L)=area(\mathrm{Int}\left( L \right))$ and the width *W*($L$) defined as the width of the thinnest band enclosing $L$ (a band being the region delimited by two parallel lines). This (minimum) width was simply estimated with the formula

$W\left( L \right)=\min_{\theta} \left( \max_{\left( x,y \right)\in L} \left( x\cos\theta+y\sin\theta\right)-\min_{\left( x,y \right)\in L} \left( x\cos\theta+y\sin\theta\right) \right)$ (5)

The biovolume of a dividing cell can be calculated with:

$V=\frac{\pi}{4}. W\left( L \right) . A(L)$ (6)

### Normalization of the signal to the cell morphology

For a centric diatom the cell in the girdle band view can be approximate to a slightly extended squared or rectangle [[16](#_ENREF_16)], with the SDVs extending essentially along the width axe. Therefore, to properly estimate the HCK-123 fluorescence per individual cell, the total fluorescence intensity at the end of the exponential phase (F_1_) or at the end of the first decay phase (F_2_), we normalized the fluorescence intensity to a disk of width (*W*), and use the calibration of the HCK-123 fluorescence (Figure S6) to obtain HCK concentration per SDV equivalent. We considered that each dividing cell contains two SDVs, and that, after local background estimation (see above estimation of the signal intensity) the HCK-fluorescence mainly accumulate inside the SDV. Even if the procedure was important to normalize the fluorescent signal considering the measured cell dimensions (*i.e*., W(L) and A(L)), the results found that the HCK signal per cell at F1 or F2 vary according to the pHe remain true independently of any normalization.

## Morphometric analyses

We started from the original image (Figure S2*A*) and then filter the image by a special Noise reduction algorithm (see denoising above) (Figure S2*B*). We can see on the figure that the pores correspond to the more intense color. Therefore, by analyzing the color histogram of the image, we can determine an appropriate threshold to binarize the image (Figure S2*C*). By using the image processing toolbox of Matlab, we can identify the circular regions (see green circles in Figure S2*D*). We can extract the radius of the identified circles as well as their positions. The radius (*R*) gives immediately statistics on the pore size. The ratio of the surfaces gives immediately the density (*ρ*).

From the positions we computed the Voronoi diagram (see blue lines in Figure S2*D*). In this latest diagram each centers of pores as a few associated neighbors. With *d* corresponds to the calculated distance between two neighbors. We can see that *d* as two main components: either d is “small” and corresponds to neighbors are in the same semi-continuous cribrum (this distance is here named *d_1_*), or d is larger and corresponds to the distance between pores that are in two different semi-continuous cribra (this distance is named *d_2_*). The area in between semi-continuous cribrum or in between pores that corresponds to dense silica is known has the hyaline area (for detailed explanation on the morphology of the valve of *Thalassiosira* species see [[17](#_ENREF_17),[18](#_ENREF_18),[19](#_ENREF_19),[20](#_ENREF_20)]). Indeed, the observation of the histogram that corresponds to measure of *d* showed a double Gaussian. We identify *d1* as the first peak and *d*2 as the second one (Figure S2*E*). From such analysis, we estimated a threshold value to determine if two neighborhood pores belong to the same or two different semi-continuous cribrum. By reconsidering all the pair of neighbors, we then estimated all the pairs of pores which are situated across two semi-continuous cribra and therefore determined new points (the middle of the segment formed by this pair) which corresponded to the middle of a radial rib. We obtained a new figure which is a morphological map of the radial ribs (Figure S2*F*).

In order to determine the distance between semi-continuous cribra, we compute a second Voronoi diagram where the Voronoi points taken are the obtained points in the radial ribs; the second Voronoi diagram is shown in Figure S2*G*. We named *D* the distance between two neighbors in this diagram. We can see that this measurement *D* has also two main components: a short component corresponding to close points in the same radial rib and a longer component corresponding to neighbors from one radial rib to the other. We filtered the results with the following algorithm: if it is possible to link two points by a succession (arbitrary chosen to 4 in our case) of paths shorter than the threshold, we define the two points as in the same radial rib, and ignore their distance. Figure S2*H* gives an example of the distances considered in the example, and Figure S2*I* gives an example of filtered results which are reasonably fitted by a Gaussian.

## Modeling

The silica polycondensation process involves several steps including transport of silicic acid into the cell, probably storage of silicic acid and/or silica, transport of silicic acid/silica into the silica deposition vesicle (SDV) and polycondensation. The latest step can be simplified to the following reaction:

$\left( SiO_{2} \right)_{n-1}+Si\left( OH \right)_{4}\begin{matrix} k_{1} \\ \rightleftharpoons\\ k_{-1} \end{matrix} \left( SiO_{2} \right)_{n}+H_{2}O$ (7)

where k are rate (kinetic) constants, and *k_1_* corresponds to the polycondensation (dehydratation) reaction and *k_-1_* corresponds to the dissolution (hydratation) reaction. Here we assume that at least inside the SDV (with acidic pH, high concentration of silicic acid and the presence of polymerizing organic molecules (namely, polyamine and sillaffin-like proteins) the reaction is essentially irreversible (*i.e.*, $k_{-1}\ll1$); in other words dissolution is not taken into consideration in our model (Figure 6). For convenience we define $\left( SiO_{2} \right)=Si$, then the reaction of silica polycondensation inside the SDV can be simplified into:

$\left( Si \right)_{n-1}+{Si}_{v}\underset{\to}{k_{1}} \left( Si \right)_{n}$ (8)

In our model we assume that *n* represents the average length of the polycondensate. Therefore, we define (Si)*_n_* the condensate, and [(Si)*_n_*] its concentration.

For simplification we also consider that the proton and silicic acid transporters are homogenously distributed around the SDV membrane. The transport and the chemical form (*i.e*., monosilicic acid, small polymeric silica or silica particles) of silicon that enters the SDV are not known. However, we assume that Si(OH)_4_ is transported by silicic acid transporters (SITs), and that the same or different SITs are involved in both the transport inside the cell and inside the SDV. Alternative hypothesis have been proposed for Si transport such as pinocytosis [[21](#_ENREF_21)], ionophore diffusion [[22](#_ENREF_22)] or silica transport vesicles (STVs) [[23](#_ENREF_23)], but none of these hypothesis have received direct evidences;they are not considered here. Protons and at least one of the buffer species (either the fluorophore, silicic acid, or both) can be electrically charged. Thus, a full description of diffusion should incorporate effects of the electric field in an electrodiffusion approach. Furthermore, if protons or silicic acid are co- or counter-transported with another substrate a gradient will be generated for the other substrate too, that in turn will influence the proton diffusion and the transport. Fortunately, in most cases, the co- or counter-transported ions are present at high concentrations, and the bulk total ion concentration is considered to be much higher than the change of the proton and silicic acid concentrations achieved by the transport activity. Therefore, we neglected here the effect of putative electrical gradients that could be caused by transport and diffusion, and a possible limitation of transport and diffusion due to counter-transported ions limitation.

According to our model (Figure 6) and to the current knowledge on Lysotracker (see above), we can consider that HCK-123 (noted F for fluorophore) concentration inside the SDV corresponds to a mobile fraction that can be protonated, and a fraction which is bound to the polymerized silica (also named biogenic silica)

$H+F_{free} \begin{matrix} k_{2} \\ \rightleftharpoons\\ k_{-2} \end{matrix} {HF}_{bound}$ (9)

${HF}_{bound}+\left( Si \right)_{n} \begin{matrix} k_{3} \\ \rightleftharpoons\\ k_{-3} \end{matrix} \left( Si \right)_{n}HF_{fixed}$ (10)

Since purified frustules remain fluorescent (see [[9](#_ENREF_9)]) we can make the reasonable assumption that the fraction of the fluorophore that is incorporated into the biogenic silica cannot dissociate$(i.e., k_{-3}\ll1)$. Dissolution is not considered in our model. For simplification we also assume that at the equilibrium the protonation of the fluorophore is rapid (*i.e.*, $K_{2}={k_{2}}/{k_{-2}}\gg1$), and the incorporation of the fluorophore during polycondensation is also rapid (*i.e.*, $K_{3}={k_{3}}/{k_{-3}}\gg1$). The steady state of the equations (8) and (9) can be represented by:

$k_{2}\left[ H \right]\left[ F_{free} \right]=k_{-2}\left[ HF_{bound} \right]$ (11)

$k_{3}\left[ HF_{bound} \right]\left[ \left( {Si}_{n} \right) \right]=k_{-3}\left[ \left( Si \right)_{n}HF_{fixed} \right]$ (12)

The total fluorophore concentration inside the SDV can be separated into three fractions: a free, a bound and a fixed fraction, therefore the total concentration reads:

$\left[ F_{total} \right]= \left[ F_{free} \right]+\left[ F_{bound} \right]+\left[ F_{fixed} \right]$ (13)

Using the steady state equations (11) and (12), we can rewrite (13) in:

$\left[ F_{total} \right]= \left[ F_{free} \right] (1+K_{2}\left[ H \right](1+K_{3}\left[ \left( {Si}_{n} \right) \right])$ (14)

Using $K_{2}\gg1$, $K_{3}\gg1$, and assuming that the concentration of the free fluorophore stays constant (buffered by the membrane properties and renewal of the medium), gives the main result of this simplified model:

$\left[ F_{total} \right]\propto\left[ H \right]\left[ \left( {Si}_{n} \right) \right]$ (15)

This suggests that in a first approximation, the value of the [HCK-123] per SDVequivalent is a good indication of the SDV proton concentration and silica accumulation.

# Additional references

1. Brzezinski MA, Conley DJ (1994) Silicon deposition during the cell cycle of *Thalassiosira weissflogii* (Bacillariophyceae) determined using dual rhodamine 123 and propidium iodide staining. J Phycol 30: 45-55.

2. Schroder HC, Perovic-Ottstadt S, Rothenberger M, Wiens M, Schwertner H, et al. (2004) Silica transport in the demosponge *Suberites* domuncula: fluorescence emission analysis using the PDMPO probe and cloning of a potential transporter. Biochem J 381: 665-673.

3. Diwu Z, Chen CS, Zhang C, Klaubert DH, Haugland RP (1999) A novel acidotropic pH indicator and its potential application in labeling acidic organelles of live cells. Chem Biol 6: 411-418.

4. Hazelaar S, van der Strate HJ, Gieskes WWC, Vrieling EG (2005) Monitoring rapid valve formation in the pennate diatom *Navicula salinarum* (Bacillariophyceae). J Phycol 41: 354-358.

5. Lin HJ, Herman P, Kang JS, Lakowicz JR (2001) Fluorescence lifetime characterization of novel low-pH probes. Anal Biochem 294: 118-125.

6. Shimizu K, Del Amo Y, Brzezinski MA, Stucky GD, Morse DE (2001) A novel fluorescent silica tracer for biological silicification studies. Chem Biol 8: 1051-1060.

7. Znachor P, Nedoma J (2008) Application of PDMPO technique in the studiing of silica deposition in natural diatom populations in a eutrophic reservoir. Journal of Phycology 44: 518-525.

8. Leblanc K, Hutchins DA (2005) New applications of a biogenic silica deposition fluorophore in the study of oceanic diatoms. Limnology and Oceanography Methods 3: 462-476.

9. Descles J, Vartanian M, El Harrak A, Quinet M, Bremond N, et al. (2008) New tools for labeling silica in living diatoms. New Phytol 177: 822-829.

10. Groger C, Sumper M, Brunner E (2008) Silicon uptake and metabolism of the marine diatom *Thalassiosira pseudonana*: Solid-state ^(29)^Si NMR and fluorescence microscopic studies. J Struct Biol 161: 55-63.

11. Vrieling EG, Gieskes WWC, Beelen TPM (1999) Silicon deposition in diatoms: control by the pH inside the silicon deposition vesicle. J Phycol 35: 548-559.

12. Louchet C, Moisan L (2010) Total variation as a local filter. SIAM J Imaging Sci 4: 651-694.

13. Rudin L, Osher S, Fatemi E (1992) Nonlinear total variation based noise removal algorithms. Physica D 60: 1-4.

14. Buades A, Coll B, Morel J-M (2005) A review of image denoising algorithms, with a new one. Multiscale Modeling and Simulation 4: 490-530.

15. Monasse P, Guichard F (2000) Fast Computation of a Contrast Invariant Image Representation. IEEE Transactions on Image Processing 9: 860-872.

16. Hillebrand H, Dürselen C-D, Kirschtel D, Pollingher U, Zohary T (1999) Biovolume calculation for pelagic and benthic microalgae. J Phycol 35: 403-424.

17. Hayashi T, Tanimura Y, Sakai H (2007) A fossil freshwater *Thalassiosira*, *T. inlandica* sp. nov. (Bacillariophyta), with semicontinuous cribra and elongated marginal fultoportulae. Phycologia 46: 353-362.

18. Kaczmarska I, Beaton M, Benoit AC, Medlin LK (2005) Molecular phylogeny of selected memebers of the order *Thalassiosirales* (Bacillariophyta) and evolution of the fultoportula. J Phycol 42: 121-138.

19. Makarova IV (1981) Principles of the systematics of *Thalassiosira* Cleve and the significance of its taxonomic characters. In: Ross R, editor. Proceedings of the 6th Symposium on Fossil and Recent Diatoms. Koeltz, Koenigstein. pp. 1-14.

20. Round FE, Crawford RM, Mann DG (1990) The diatoms, Biology and Morphology of the Genera. Cambridge: Cambridge University Press.

21. Vrieling EG, Sun Q, Tian M, Kooyman PJ, Gieskes WW, et al. (2007) Salinity-dependent diatom biosilicification implies an important role of external ionic strength. Proc Natl Acad Sci USA 104: 10441-10446.

22. Thamatrakoln K, Kustka AB (2009) When to say when: can excessive drinking explain silicon uptake in diatoms? Bioessays 31: 322-327.

23. Schmid A-MM, Schulz D (1979) Wall morphogenesis in diatoms: deposition of silica by cytoplasmic vesicles. Protoplasma 100: 267-288.
